# Variation in the morphology of spinous processes in the cervical spine – An objective and parametric assessment based on CT study

**DOI:** 10.1371/journal.pone.0218885

**Published:** 2019-06-27

**Authors:** Kamil Ludwisiak, Michał Podgórski, Katarzyna Biernacka, Ludomir Stefańczyk, Łukasz Olewnik, Agata Majos, Michał Polguj

**Affiliations:** 1 Department of Diagnostic Imaging of the Central Clinical Hospital of the Medical University of Lodz, Lodz, Poland; 2 Department of Diagnostic Imaging, Polish Mother's Memorial Hospital Research Institute, Lodz, Poland; 3 Department of Radiology and Diagnostic Imaging, Medical University of Lodz, Lodz, Poland; 4 Department of Normal and Clinical Anatomy, Interfaculty Department of Anatomy and Histology, Medical University of Lodz, Lodz, Poland; 5 Department of Angiology, Interfaculty Department of Anatomy and Histology, Medical University of Lodz, Lodz, Poland; The Cyprus Institute, CYPRUS

## Abstract

**Background:**

Typically, cervical vertebrae display bifid spinous processes. Nevertheless, this feature may vary both between subjects and even within the vertebrae of the same individual. Although such variation can be important in archaeological research, anthropological studies and forensic medicine, it has not so far been the subject of any detailed studies.

**Material and methods:**

An analysis of 200 cervical spine CT examinations was performed. The morphology of the spinous process was evaluated, and new anthropometric parameters were selected to allow a more precise quantitative analysis of the degree of bifidity.

**Results:**

The spinous process base (i.e. the part of the spinous process which was not bifid) was significantly longer in CII and CVII than in the other vertebrae. The spinous process branches (bifid elements) were significantly longer in CVI and CVII than in the other vertebrae. The angle between the branches was significantly sharper in CII and CVII than in CIII-CVI, on the right side, and CIII-CV, on the left side. On the right side, the branching coefficient (degree of branch development) was significantly higher for CII and significantly lower for CVI-CVII than for the other vertebrae. On the left side, the coefficient was significantly higher for CII and CIV, and significantly lower for CVI-CVII, compared to the other vertebrae.

**Conclusion:**

Our findings highlight new objective parameters of morphological variability in the spinous processes of the cervical spine. They can form the basis of a new detailed differentiation of vertebrae and can represent an independent determinant of anatomical variability in the cervical spine.

## Background

**Fig 1:**
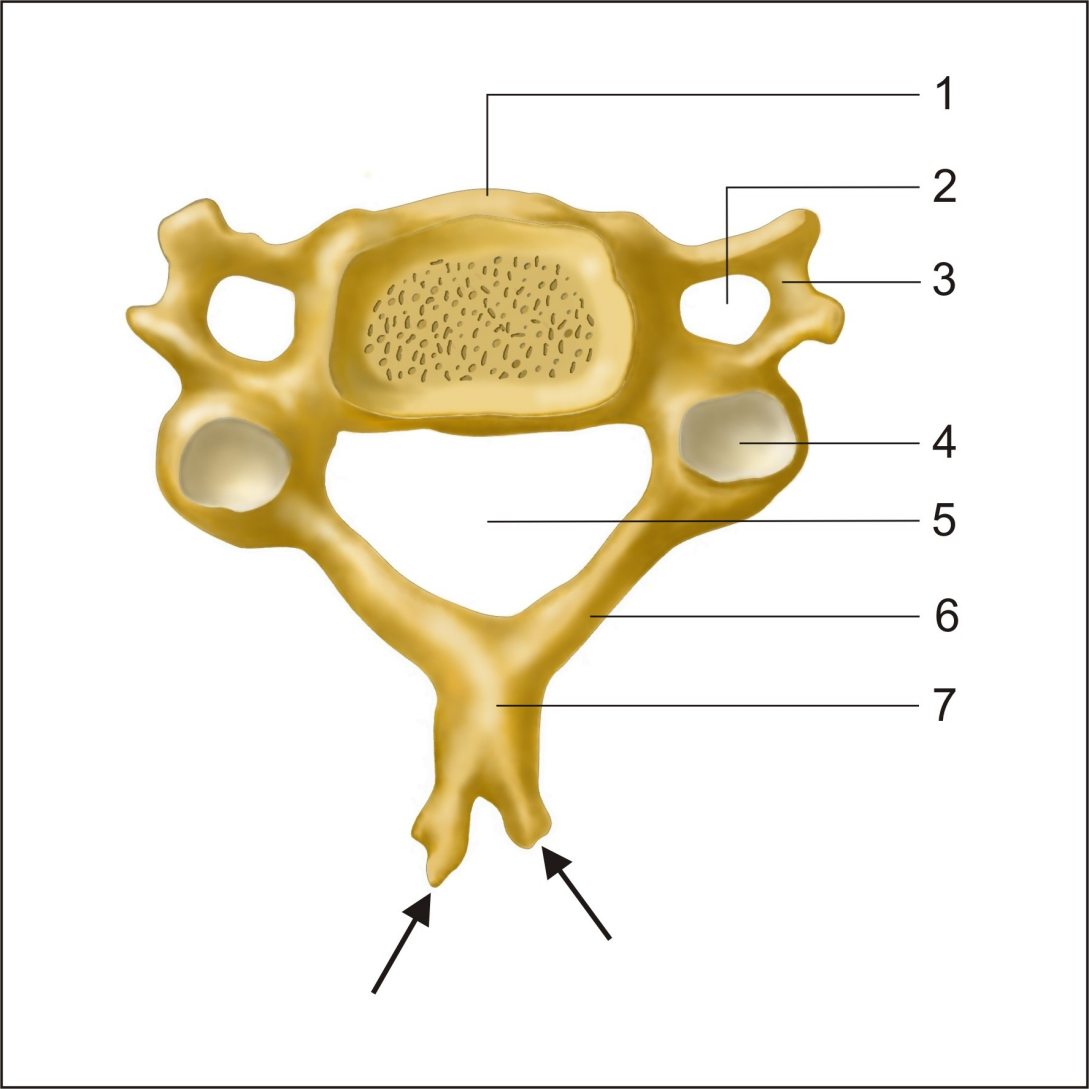
Schematic arrangements of typical cervical vertebra. 1—vertebral body, 2—foramen transversarium, 3—transverse process, 4—superior articular process, 5—vertebral foramen, 6—vertebral arch, 7—spinous process, arrows—bifurcation of spinous processes.

**Fig 2:**
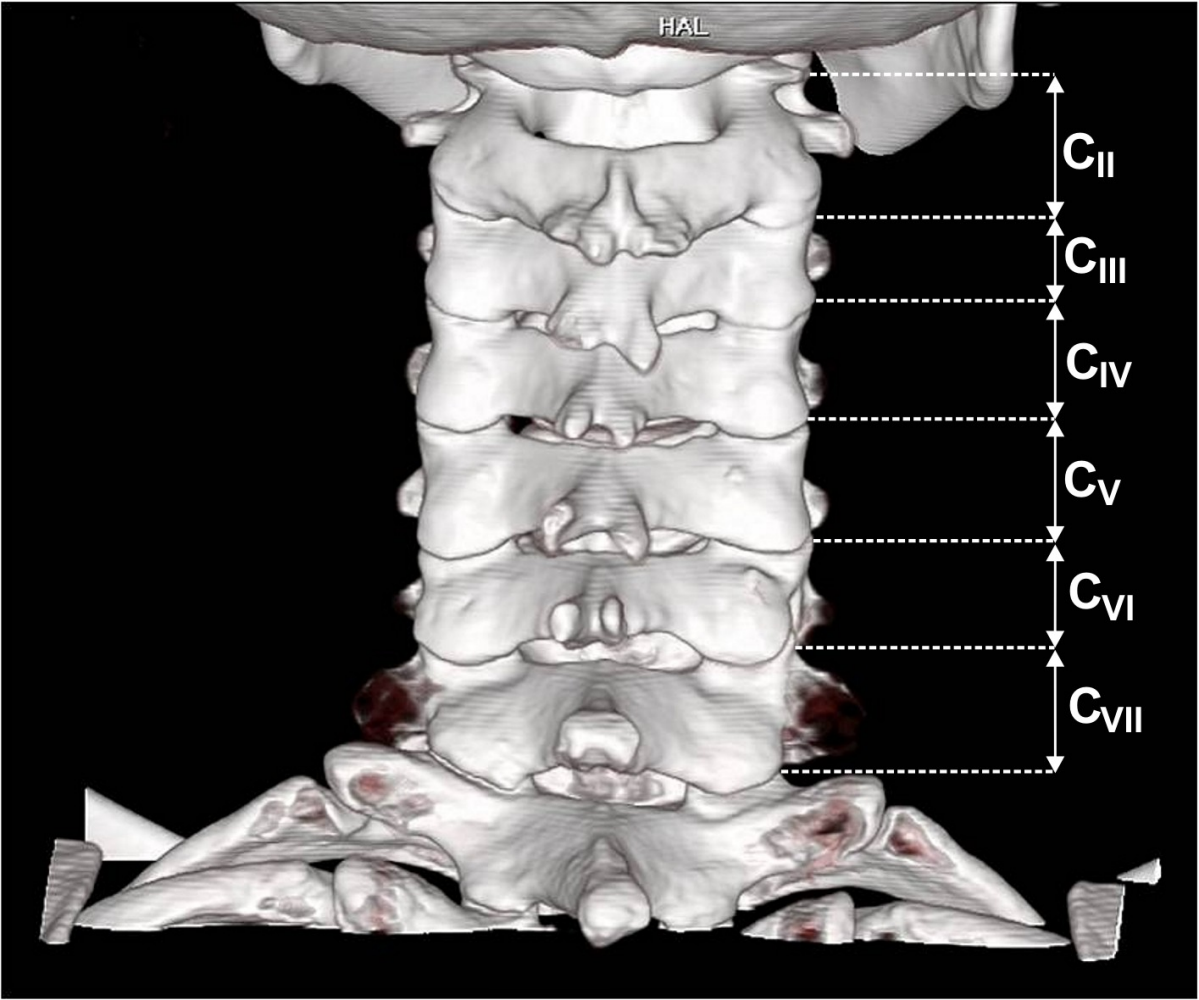
Three-dimensional volume rendering (VR) CT demonstrating bifid spinous processes in the cervical spine, level CII-CVII. Multidetector Computed Tomography (MDCT).

Most previous works describe bifurcation of the cervical spinous processes as a characteristic feature that can be seen most clearly in vertebrae CIII to CVI (Figs 1 and 2) [1]. However, some 20th century publications recognize it as a developmental variant [2] that can be associated with sex and race [3].

Despite its potential importance in anthropological [4] or even forensic [3,5] studies, no detailed data on variation in the degree of spinous process bifurcation can be found in the published literature. This may be because most existing studies do not specify the research methodology or employ subjective methods: for example, Shore and Duray [5,6,7]. Therefore, it is necessary to create an original method for analyzing the variation in the morphology of spinous processes of the cervical vertebrae based on an objective morphometric measurement and statistical analysis. Our study provides one of the first such detailed descriptions and quantitative analyses of the morphology of the spinous processes in the cervical spine. It is of potential use in archeology, anthropometric studies, forensic medicine [8] and comparative anatomy [9].

## Material and methods

A retrospective analysis was performed of CT examinations of the cervical spine carried out between 2014 and 2018. An on-line random date generator was used (http://random-date-generator.com) to select days from this period and all examinations from a given day were analyzed. All patients included in the analysis were from Poland, Central Europe. The protocol of the study was accepted by the Local Bioethical Commission of the Medical University of Lodz (Resolution no. RNN/299/16KE).

All the examinations were performed with a 128-line Siemens Sensation scanner. Images were acquired according to a predefined protocol for examination of the cervical spine which performs spiral scans every 1 mm, and saves the obtained images in DICOM format with a B70 bone filter.

The inclusion criterion was a correctly-performed tomographic examination with the patient in the supine position; the position of the scapulae was irrelevant, but with the head in a neutral position, as suggested by Otsudo et. al. [10]. The exclusion criteria comprised cervical spine injury (e.g. fractures), vertebral deformation (e.g. due to massive osteophytosis), presence of malignancies with bone metastases and examinations containing artifacts that prevent an analysis of the cervical spine from CII to CVII.

**Fig 3:**
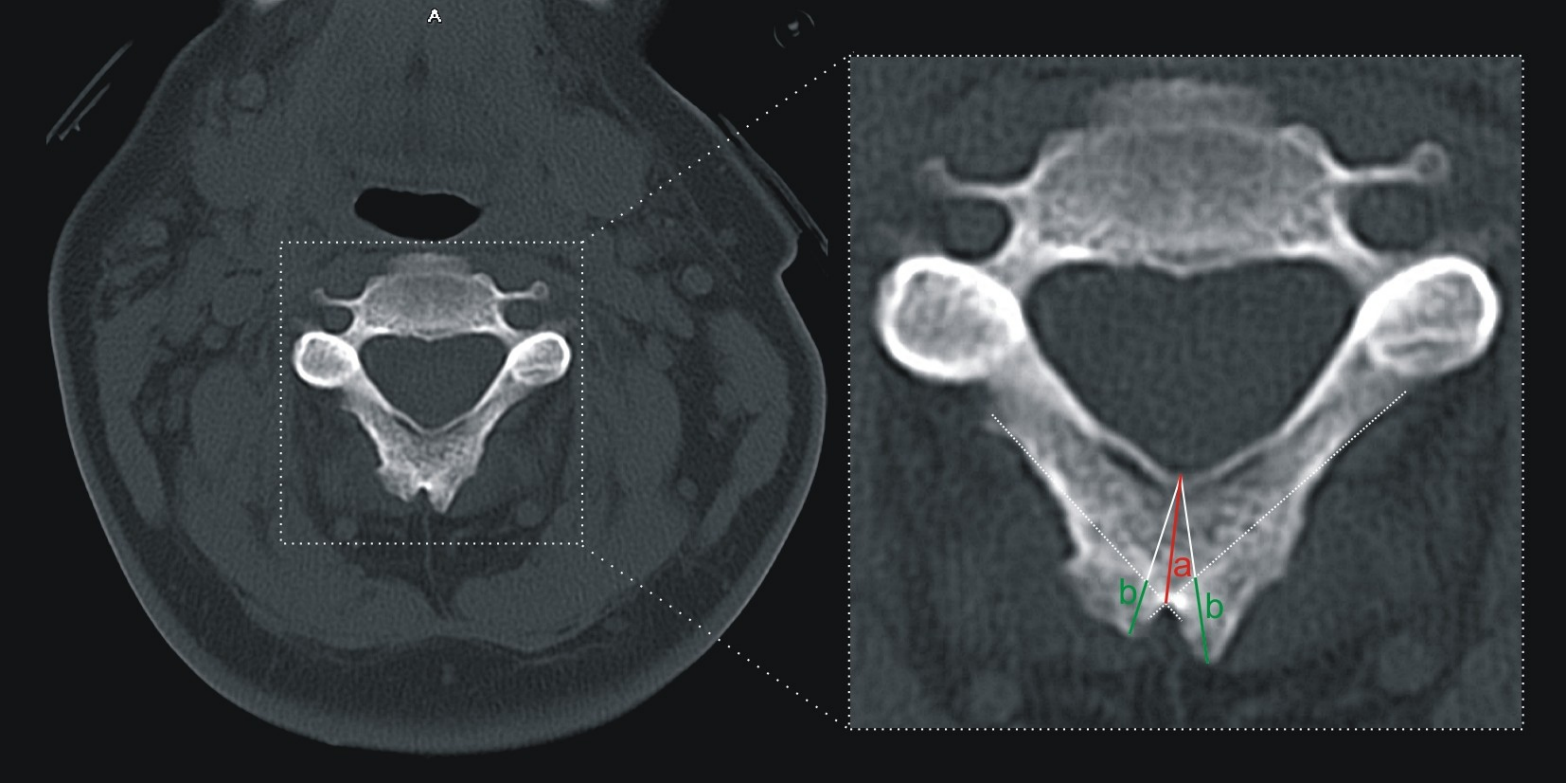
Defined and measured dimensions of the spinous processes. a—length of the spinous process base; b—length of the spinous process branch. Multidetector Computed Tomography (MDCT).

**Fig 4:**
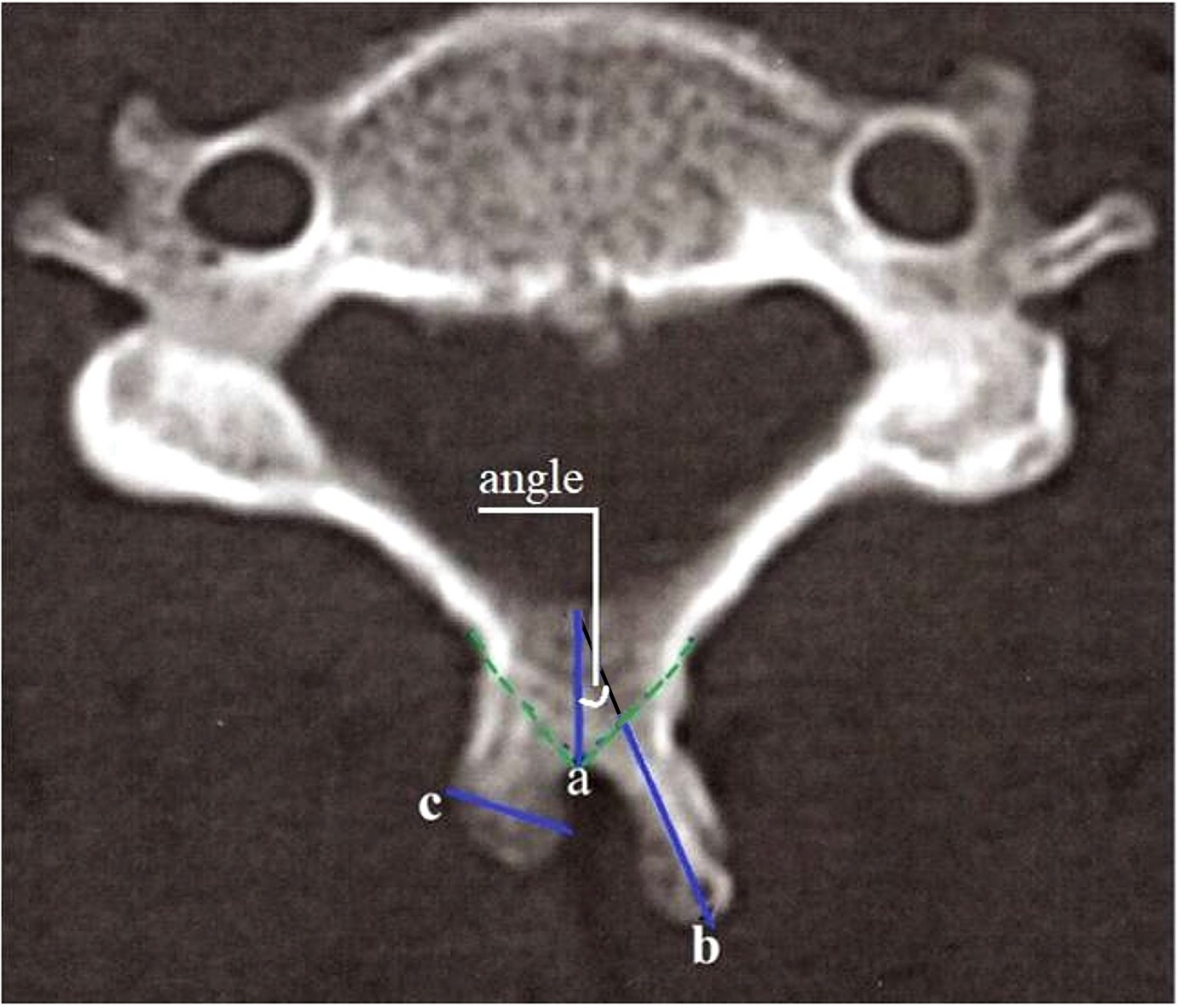
Defined and measured dimensions of the spinous processes–a zoomed view. a—length of the spinous process base; b—length of the spinous process branch; c—width of the spinous process branch; “angle”–branching angle. Multidetector Computed Tomography (MDCT).

For data analysis the native console (SyngoVia, Siemens) was applied. The short axis of each vertebra from CII to CVII was obtained by scan reconstruction and by adjusting the examination planes for each vertebra. Therefore, the presence of any dorso-inferior inclination of the vertebrae due to lordosis did not bias any measurements. To determine the exact dimensions of the spinous process and to characterize the morphology of bifurcation, the following parameters were used (Figs 3 and 4):

- Length of the spinous process base (a)–the shortest distance from the most posterior point of the vertebral bony central canal to the deepest point between the two branches of the spinous process (Figs 3 and 4).
- Length of the spinous process branch (b)–first, for each branch a line is drawn connecting the most posterior point of the vertebral bony central canal to the tip of the branch. Then the line contingent to the external surface of the lamina of the vertebral arch is marked. The length of the spinous process branch is determined as a distance from the crossing point of the aforementioned lines to the tip of the spinous process branch (Figs 3 and 4).
- Branching angle–the angle between lines *a* and *b* (deviation from the sagittal axis of the spinous process) (Fig 4).
- Width of the spinous process branch—the maximum width of the spinous process measured in the axis perpendicular to line *b* (c) (Fig 4).

Based on the described measurements, we propose a new parameter that characterizes the degree of development of the spinous process–the *branching coefficient*. It is calculated for each branch by dividing distance *a* by distance *b*, described above, and multiplying the obtained result by 100%.

The statistical analysis was performed with Statistica 12.0 software (StatSoft Polska. Cracow. Poland). A p-value lower than 0.05 was considered significant.

The normality of the continuous data distribution was verified by the Shapiro-Wilk test. As the data was not normally distributed, the Mann-Whitney test and the Wilcoxon sign-rank test were used to compare the anthropometric measurements between the sexes and body sides, respectively. The Friedman ANOVA with a dedicated *post hoc* test was used to compare these measurements between the vertebrae.

## Results

Of the whole number of 251 chosen examinations, 200 were eligible to enter the study (101 females, 99 males, the mean age 42, SD 17).

No significant differences were observed between the sexes with regard to any of the analyzed parameters; however, all tested parameters except for the branching coefficient differed significantly between body sides (Table 1). Therefore, they were compared separately in the following analysis.

**Table 1:** Comparison of the morphological parameters between body sides.

Parameter	Body side	Mean	SD	p-value
Length of the spinous process branch[mm] (b line)	R	1.07	0.45	0.0034
	L	1.10	0.49	
Branching angle[°]	R	13.45	6.76	0.0002
	L	13.19	6.43	
Branching coefficient[%]	R	95.53	79.72	0.3409
	L	93.09	93.77	
Width of the spinal spinous process branch[mm]	R	0.40	0.16	0.0029
	L	0.43	0.48	

**Fig 5:**
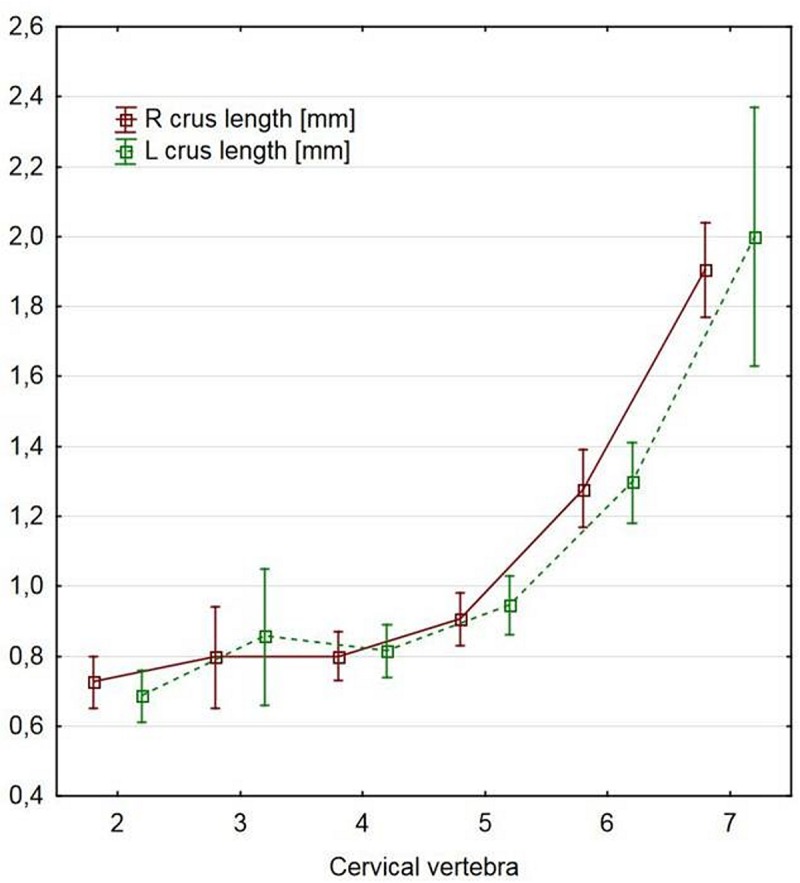
Differences in the length of the spinous process branch between the vertebrae.

The spinous process base was significantly longer in CII and CVII than in the other vertebrae (p<0.0001, Table 2). The spinous process branches were significantly longer in CVI and CVII than in the other vertebrae (p<0.0001; Table 2, Fig 5).

**Table 2:** Morphological parameters of the spinous processes. Data presented as a mean (SD).

Level	Body side	Length of the spinous process peduncle (a)[cm]	Length of the spinous process branch[mm] (b line)	Branching angle[°]	Width of the spinous process branch[mm]	Branching coefficient (a/b x 100%)
CII	R	1.08 (0.32)	0.73 (0.28)	12.5 (6.56)	0.42 (0.16)	177.4 (111.3)
	L		0.69 (0.28)	11.8 (6.36)	0.42 (0.13)	121.7 (92.3)
CIII	R	0.67 (0.22)	0.80 (0.53)	13.8 (8.20)	0.40 (0.17)	92.4 (67.8)
	L		0.86 (0.71)	14.0 (7.23)	0.40 (0.16)	90.1 (60.9)
CIV	R	0.62 (0.16)	0.80 (0.27)	15.3 (7.79)	0.39 (0.13)	95.5 (47.2)
	L		0.81 (0.29)	15.0 (7.45)	0.50 (1.13)	143.8 (82.0)
CV	R	0.63 (0.20)	0.91 (0.29)	14.0 (6.12)	0.36 (0.13)	89.7 (63.6)
	L		0.95 (0.31)	14.2 (6.04)	0.38 (0.14)	82.1 (70.7)
CVI	R	0.70 (0.22)	1.28 (0.41)	13.5 (5.54)	0.37 (0.12)	72.1 (66.8)
	L		1.30 (0.43)	12.5 (4.99)	0.39 (0.11)	74.1 (70.7)
CVII	R	1.02 (0.33)	1.90 (0.49)	11.6 (5.31)	0.46 (0.22)	46.1 (28.2)
	L		2.00 (1.30)	10.9 (5.18)	0.47 (0.24)	46.8 (29.4)

*R—right*. *L–left*

**Fig 6:**
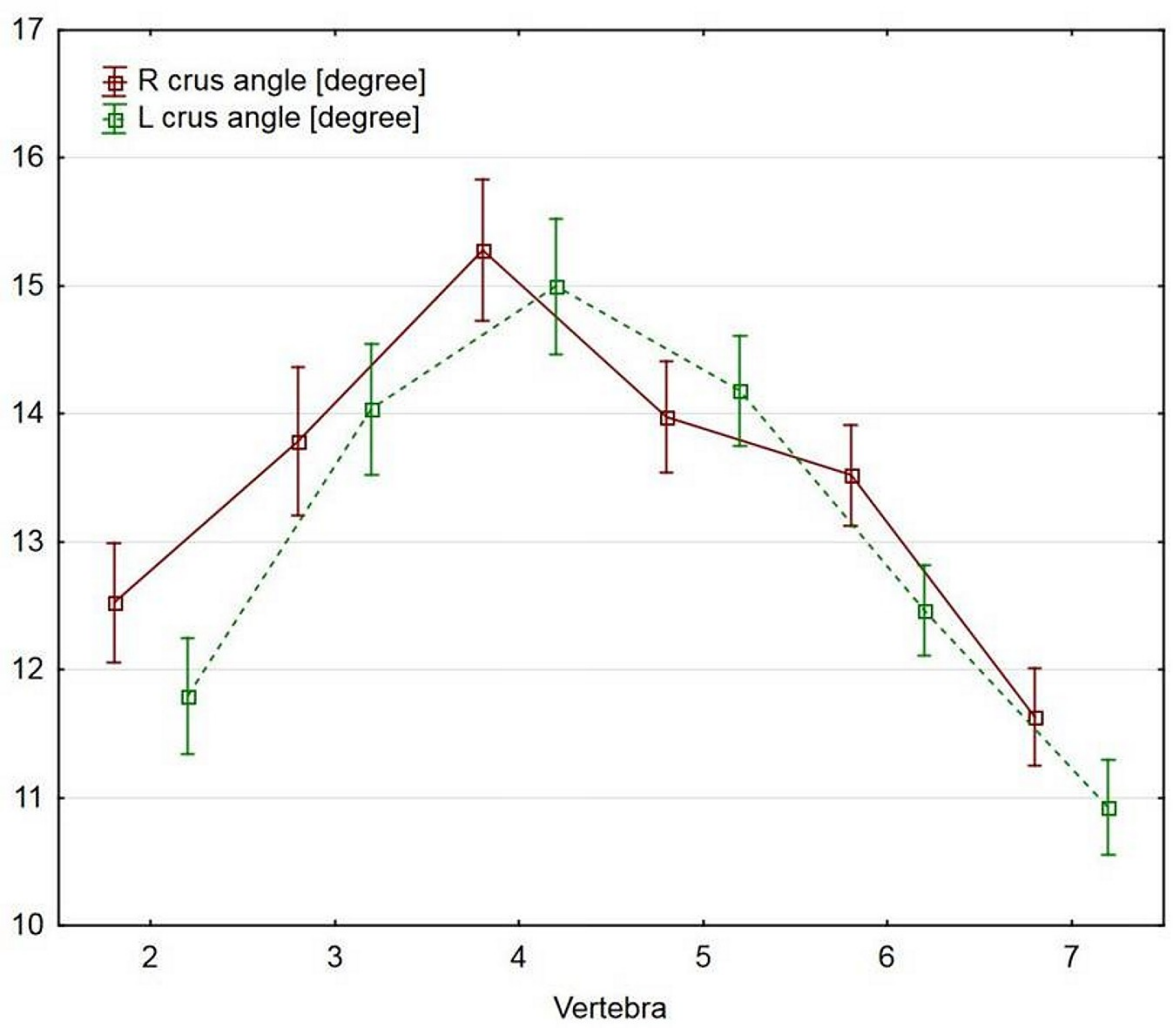
Differences in the branching angle between vertebrae.

In all bifurcated spinous processes, the branching angle was significantly sharper in CII and CVII than in the CIII-CVI vertebrae, on the right side, and CIII-CV, on the left side (p<0.0001; Table 2, Fig 6). The maximum branch width did not differ significantly between the vertebrae (p = 0.5964 for the right side, and p = 0.5956 for the left).

**Fig 7:**
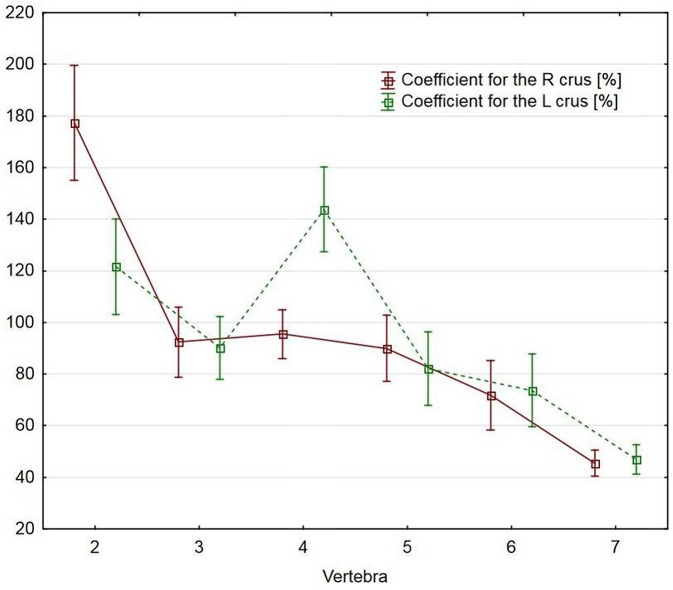
Differences in the branching coefficient between the vertebrae.

On the right side, compared to the other vertebrae, the branching coefficient was significantly higher for CII and significantly lower for CVI-CVII (p<0.0001). Similarly, on the left side, the coefficient was significantly higher for CII and CIV and significantly lower for CVI-CVII (p<0.0001; Table 2, Fig 7).

A detailed analysis of the differences in the morphological parameters between the vertebrae is presented in Table 2.

## Discussion

The most commonly-used methods employed to evaluate cervical spine bifurcation are anthropological analysis of the human skeleton [2,3,5,8], anatomical dissection [11], and imaging studies of living subjects with X-ray or CT [7,12, 13]. Our present work, based on CT examination, developed objective anthropometric measurements for the quantitative assessment of the degree of the spinous process bifidity. The proposed classification is based on variance in the morphology of the common part of the spinous process and its branches, i.e. their length and pattern of branching.

Our coefficients allow precise examination to be made of the spinous processes. Such characterization is very important because, according to Greiner [14], cervical vertebrae with shorter spinous processes tend to display a more pronounced bifid condition.

Previous classifications tended to be simpler. Shore and Duray distinguish three types of bifidity: full, partial or lack of bifurcation [5,6], while Cho et al. propose a tripartite classification based on anatomical and CT study: lack of bifurcation, partial bifurcation or full bifurcation [7]. Our proposed classification is more accurate and less subjective because its criteria are based on quantitative morphometric measurements suggested *inter alia* by Zhang et al. [13], dealing with morphometric measurements of the CVII spinous processes, which also suggests a proper methodology for performing measurements based on 3D CT scans.

Such precise features are required before reliable studies on anatomical variations can be performed according to population and gender [15]. Stephen et al. [5] report only a slight sexual dimorphism, in which full bifurcation of the spinous process dominated among men and partial bifurcation among women, with this dimorphism being most pronounced in an African population. Other parameters, such as the length of spinous processes, did not differ significantly [5]. These results contrast with our current findings, i.e. no significant sexual dimorphism was observed, and those of Zhang et al. [13] on a Chinese population, in which no statistically significant differences were observed in age or male to female ratio. Although this difference may be due to variation between populations, it could also be influenced by the fact that a more detailed evaluation was made possible by the use of more subtle, objective and continuous descriptors of this feature.

In studies on European populations, full bifurcation of the spinous process has been found to be more common at the levels CIII to CVI [6,9]. However, Allbrook [16] reports that the bifid process occurred most commonly in CII and CV. In our study, it was also present at CII. However, any direct comparison with our results is complicated by the fact that none of the previous publications analyzed the branching coefficient. Such comparison will be easier in future studies following unification of the determinants describing the degree of bifurcation.

Some studies in European and Native African populations have reported a higher incidence of bifid spinous processes in foetuses than in adults [6]. However, sometimes unbifided spinous process have been observed in the third, fourth and sixth cervical vertebrae [17].

Our proposed scheme for the objective evaluation of bifidity could be of value in clinical anatomy [15, 18, 19, 20], forensic anthropology [4] and court cases, as well as in the analysis of human remains [3]. As suggested by Kocabiyik et al. [15], “the use of more accurate morphometric measurements on the entire cervical spine and their comparison with work on similar analyzes, as well as the study of correlation, significance level and other features of this area will allow to obtain more accurate conclusions on dependencies in the population and sexual dimorphism”, also in the occurrence and types of spinous process bifurcation in the cervical spine [21]. Furthermore, the presented method is universal and it can be transferred to populations representing different time periods and different cultures. In clinical anatomy, the parameters allow standardization and may be further used in research related to pathological conditions, such as analyses of bone defects or posture disorders, e.g. cervical kyphosis. This may translate into better and more precise methods of treatment [3].

The main limitation of our work is the narrow population that was examined. However, the purpose of this research was not to evaluate a broad spectrum of variability in a population but to create a tool that can be further validated and applied for other populations. Another limitation of the work was the paucity of information about the subjects. Apart from basic details, such as sex, age, origin, and skin color, no other information which could affect the type of musculoskeletal structures in the studied area, such as patient weight, height, lifestyle or physical activity, could be obtained. Nevertheless, as mentioned above, we did not intend to explain any factor that might be responsible for the anatomical variation of the spinous processes; however, we hope that with the methodology developed herein, this may serve as the aim of our next study.

## Conclusion

The use of objective, quantitative parameters based on morphometry allows for accurate characterization of spinous process bifidity in the cervical region. Due to the multiparametric character of this classification, these parameters are independent factors characterizing vertebral morphology.

## Supporting information

S1 Table Additional file 1. (PDF)
