# Supplementary material for: Variation in the morphology of spinous processes in the cervical spine – An objective and parametric assessment based on CT study
Source: PLoS One. 2019 Jun 27;14(6):e0218885. doi: 10.1371/journal.pone.0218885 (PMC6597074; doi:10.1371/journal.pone.0218885)
Supplement: S1 Table — (PDF) [file pone.0218885.s001.pdf]

Legend
l.p. - no of patient
Date – date of examination in RRRR.MM.DD format
Gender: 0=Female / 1=Male
Age: w latach
1. Length of the spinous process base (a) [cm]
2. Length of the spinous process branch (b) [cm]
3. Branching angle – the angle between "a" and "b" lines [degrees]
4. The branching coefficient [%]
5. Width of the spinous process branch [cm]
C 1 – 7: level of cervical spine
R – Right
L – Left

l.p.	Patient			1									
	Date	Gender	Age	C2	C3	C4	C5	C6	C7	C2 R	C2 L	C3 R	C3 L
1	2014.12	0	23	0,85	0,46	0,66	0,83	0,81	0,86	0,58	0,82	1,05	0,64
2	2014.12	1	24	0,71	0,20	0,41	0,37	0,55	0,64	0,60	0,98	0,92	1,10
3	2014.12	1	43	1,09	0,94	0,37	0,52	0,62	0,93	1,07	0,79	0,64	0,42
4	2015.01	1	57	1,17	0,67	0,46	0,88	0,45	0,76	0,96	0,87	1,49	1,47
5	2015.01	1	24	0,72	0,54	0,52	0,32	1,22	1,47	0,44	0,59	0,79	0,82
6	2015.01	0	32	1,10	0,53	0,59	0,38	0,82	0,89	0,48	0,36	0,38	0,38
7	2015.02	0	37	0,70	0,97	0,75	0,68	0,60	0,99	0,52	0,49	0,35	0,23
8	2015.02	0	73	1,23	0,46	0,69	0,45	0,86	1,10	0,62	0,56	0,73	1,25
9	2015.02	0	87	0,95	0,72	0,71	0,60	0,46	0,61	0,69	0,57	0,77	0,81
10	2015.02	1	75	0,72	0,91	0,72	0,65	0,74	0,91	0,47	0,21	0,11	1,02
11	2015.03	1	28	1,73	0,65	0,57	0,37	0,47	0,70	0,20	0,57	0,71	0,80
12	2015.03	1	33	0,85	0,56	0,48	0,73	0,70	0,80	0,48	0,49	0,51	0,42
13	2015.04	1	33	0,88	1,07	0,59	0,72	0,59	1,58	0,62	0,59	0,20	0,29
14	2015.04	1	20	1,46	0,84	0,52	0,49	0,82	1,42	0,74	0,36	0,64	0,43
15	2015.05	0	30	0,85	0,53	0,52	0,52	0,39	0,89	0,59	0,49	0,94	0,63
16	2015.05	0	48	0,72	0,68	0,83	0,73	0,65	1,42	0,41	0,83	0,42	0,69
17	2015.05	1	30	0,62	0,72	0,79	0,82	0,84	1,16	0,43	0,12	0,23	1,04
18	2015.05	1	25	0,93	0,77	0,75	0,57	0,64	0,71	0,64	0,50	0,85	0,97
19	2015.06	0	75	0,89	0,46	0,42	0,48	0,60	0,76	0,44	0,60	0,18	0,46
20	2015.06	0	27	0,97	1,10	0,76	0,97	0,76	0,64	0,36	0,35	0,46	0,38
21	2015.06	1	27	0,57	0,64	0,75	0,45	0,52	1,14	0,38	0,45	0,63	0,67
22	2015.07	1	69	1,22	0,46	0,53	0,73	0,58	1,02	0,99	0,54	0,90	1,01
23	2015.07	0	55	1,06	0,71	0,56	0,48	0,62	0,67	0,69	0,66	0,62	0,46
24	2015.07	1	76	0,72	0,88	0,65	1,00	1,70	0,56	0,85	0,82	1,00	0,75
25	2015.07	1	61	0,85	0,64	0,58	0,66	0,72	1,08	0,67	0,85	0,00	1,00
26	2015.09	0	29	0,57	0,42	0,42	0,39	0,58	1,21	0,52	0,27	1,04	0,62
27	2015.09	1	59	0,88	0,40	0,46	0,43	0,69	0,84	0,75	0,53	0,98	1,04
28	2015.10	1	28	0,44	0,63	0,57	0,68	0,41	0,99	0,35	0,24	0,67	0,79
29	2015.10	1	26	0,89	0,58	0,54	0,73	0,84	1,05	0,77	0,87	0,49	0,49
30	2015.10	0	63	1,22	0,50	0,66	0,46	0,37	1,03	0,67	0,38	0,93	0,76
31	2015.10	1	31	0,69	0,91	0,64	0,69	0,46	0,81	0,52	0,33	0,36	0,86
32	2015.10	1	85	0,10	0,67	0,51	0,52	0,63	0,61	1,58	0,30	0,40	0,74
33	2015.10	0	57	0,41	1,02	0,64	0,66	0,53	0,83	1,39	0,20	0,46	0,72
34	2015.10	0	37	0,74	0,56	0,44	0,64	0,72	0,90	0,79	0,49	0,90	0,86
35	2015.11	0	30	1,40	0,38	0,41	0,39	0,60	0,54	0,28	0,29	0,36	0,71
36	2015.11	1	28	0,64	0,62	0,46	0,56	0,59	0,60	0,35	0,85	0,67	0,92

37	2015.12	0	37	0,80	0,67	0,72	0,45	0,65	0,74	0,49	0,51	0,65	0,57
38	2015.12	1	63	0,73	0,95	0,66	0,52	0,82	1,54	0,91	1,02	0,25	0,37
39	2015.12	1	19	0,66	0,67	0,53	0,46	0,67	1,19	0,39	0,53	0,78	0,67
40	2016.01	1	27	1,24	1,10	0,70	0,74	0,52	1,08	1,17	0,95	1,28	1,34
41	2016.02	1	25	1,92	0,71	0,65	0,94	1,15	1,81	0,93	0,97	0,71	0,35
42	2016.02	1	26	1,17	0,83	0,67	1,78	0,89	1,31	0,66	0,70	0,66	0,68
43	2016.02	0	64	1,35	0,48	0,62	0,89	0,82	1,30	0,93	0,98	0,64	0,91
44	2016.02	1	33	1,29	0,51	0,50	0,23	0,72	1,34	0,57	0,58	0,63	0,90
45	2016.02	1	58	1,19	0,76	0,70	0,85	0,94	1,41	0,81	0,64	0,61	0,91
46	2016.02	1	37	1,29	0,70	0,76	0,73	0,71	1,44	1,28	0,69	1,01	0,94
47	2016.03	0	65	1,73	0,69	0,68	0,68	0,90	1,38	0,69	0,43	1,00	0,92
48	2016.03	0	43	1,13	0,62	0,61	0,54	0,46	0,63	0,76	0,91	0,76	0,34
49	2016.03	1	20	1,27	0,45	0,49	0,52	0,68	1,01	1,00	0,77	1,18	0,82
50	2016.03	0	43	0,68	0,56	0,77	0,60	0,83	1,46	0,90	0,38	1,57	1,41
51	2016.04	1	39	1,03	0,56	0,80	0,66	0,83	1,23	1,26	0,98	0,00	0,92
52	2016.04	1	50	1,18	0,79	0,90	0,85	1,07	1,14	1,26	0,46	0,63	0,89
53	2016.04	1	41	1,59	1,01	1,00	0,87	1,20	1,73	0,71	0,85	1,00	0,56
54	2016.04	1	38	1,24	1,23	1,14	0,79	0,99	1,41	1,25	1,15	1,02	1,20
55	2016.04	1	37	0,78	0,82	0,82	0,72	0,75	1,44	1,01	0,99	0,80	1,37
56	2016.04	0	19	1,01	0,57	0,57	0,56	0,80	1,01	0,90	0,90	0,81	0,45
57	2016.04	1	61	1,00	0,75	0,79	0,60	0,78	0,68	0,65	1,07	0,84	0,42
58	2016.05	1	38	0,46	0,37	0,65	0,65	0,32	0,52	0,26	0,65	0,81	0,90
59	2016.05	0	28	0,94	0,62	0,64	0,37	0,62	0,92	0,88	1,11	1,20	1,13
60	2016.05	0	33	1,24	0,67	0,63	0,66	0,51	1,29	0,66	0,65	0,78	0,86
61	2016.05	1	35	1,12	0,54	0,65	0,63	0,62	0,91	0,65	0,80	0,44	0,98
62	2016.05	0	48	0,95	0,81	0,72	0,66	0,96	1,39	0,78	0,54	0,65	0,00
63	2016.05	1	33	1,77	0,93	0,93	0,68	0,61	1,05	0,61	0,74	1,22	1,13
64	2016.05	1	39	1,03	1,09	0,80	1,28	0,93	0,64	0,31	0,46	0,82	0,82
65	2016.05	1	40	1,09	0,87	0,69	0,79	1,16	1,49	1,32	1,34	1,15	1,21
66	2016.05	0	70	1,05	0,64	0,76	0,63	0,99	0,98	0,62	0,47	0,84	1,15
67	2016.05	0	57	0,68	0,42	0,46	0,56	0,60	0,70	0,80	0,69	0,29	0,29
68	2016.06	0	65	0,98	0,78	0,70	0,74	0,79	1,44	0,82	1,08	0,82	0,92
69	2016.06	0	36	0,96	1,04	1,25	1,20	1,07	1,34	0,55	0,57	0,91	1,04
70	2016.06	0	26	1,60	0,74	0,74	0,72	0,69	0,89	0,55	0,37	0,55	0,64
71	2016.07	1	34	1,15	0,51	0,48	0,50	0,57	1,31	1,06	0,82	1,01	0,88
72	2016.07	0	56	0,97	0,72	0,88	0,65	1,00	1,70	0,56	0,85	0,82	1,00
73	2016.07	0	26	0,72	1,05	0,90	0,48	0,69	1,02	0,48	0,26	0,72	0,42
74	2016.07	1	27	1,04	0,96	0,84	0,86	0,82	1,14	0,66	0,52	0,84	0,90
75	2016.07	1	33	1,12	0,79	0,64	0,93	0,81	1,40	0,91	0,70	0,89	0,87
76	2016.08	1	27	1,33	0,57	0,51	0,79	0,75	1,25	1,33	0,90	4,09	3,85
77	2016.08	0	49	0,85	0,64	0,58	0,66	0,72	1,08	0,67	0,85	0,00	1,00
78	2016.09	1	43	1,29	0,80	1,09	0,67	0,90	1,94	0,95	0,71	0,97	0,96
79	2016.09	1	20	0,61	0,74	0,82	1,06	0,98	1,55	0,20	0,20	0,87	0,60
80	2016.10	1	26	0,97	0,98	0,65	0,75	0,75	1,68	1,03	0,63	0,76	0,86
81	2016.10	1	28	0,56	0,52	0,56	0,59	0,91	1,64	0,89	0,57	0,90	1,14
82	2016.10	0	49	1,37	0,60	0,61	1,10	0,88	1,49	0,76	0,76	1,16	0,97
83	2016.10	0	33	0,96	0,46	0,80	0,64	0,74	1,02	0,63	0,55	0,77	0,53
84	2016.10	0	64	1,13	0,78	0,65	0,93	1,12	1,17	0,76	0,72	0,85	0,87
85	2016.10	0	23	0,72	0,76	0,62	0,57	0,81	1,18	0,79	0,84	0,77	0,80
86	2016.10	1	19	1,36	0,82	0,63	0,54	0,98	1,35	1,09	1,11	0,71	1,01
87	2016.10	1	69	1,18	0,64	0,77	0,64	0,86	1,29	0,94	1,02	0,81	0,98
88	2016.10	0	25	0,89	0,72	0,68	0,90	0,94	1,13	0,88	0,55	1,01	1,10
89	2016.11	1	68	1,10	0,54	0,69	0,73	0,81	1,08	1,02	1,27	6,18	9,35

90	2016.11	0	29	1,09	0,49	0,41	0,33	0,60	0,83	0,61	0,83	0,87	0,76
91	2016.11	0	49	0,76	0,59	0,60	0,43	0,63	1,15	0,75	0,73	0,41	0,79
92	2016.11	1	45	0,72	0,59	0,51	0,60	0,48	1,40	0,16	0,55	0,97	0,58
93	2016.11	0	43	0,97	0,62	0,60	0,67	0,64	1,36	1,02	1,06	0,43	0,00
94	2016.11	0	42	0,88	0,75	0,69	0,80	1,25	1,33	1,60	1,57	1,15	1,15
95	2016.11	0	34	1,00	0,62	0,68	0,70	0,81	1,15	0,89	0,79	0,62	0,58
96	2016.11	1	19	1,72	0,43	0,51	0,60	0,65	0,95	0,72	0,70	0,78	0,96
97	2016.11	0	47	1,02	0,64	0,66	0,53	0,83	1,39	0,20	0,46	0,72	0,48
98	2016.12	0	59	1,30	0,75	0,66	0,69	0,94	1,23	0,81	0,75	0,83	0,96
99	2016.12	0	31	0,88	0,47	0,74	0,85	0,71	0,94	1,18	1,15	0,85	1,01
100	2016.12	1	20	0,77	1,06	0,79	0,86	0,66	1,43	1,45	1,22	0,89	0,69
101	2016.12	0	35	1,15	0,97	0,75	0,90	1,32	2,65	1,09	1,20	1,56	1,69
102	2016.12	0	36	1,10	0,68	0,67	0,96	0,68	1,31	0,95	1,01	0,77	0,63
103	2016.12	0	37	1,01	0,81	0,74	0,57	0,79	0,82	0,74	0,75	0,83	0,56
104	2016.12	1	20	1,38	0,79	0,59	0,61	0,58	1,06	0,66	0,74	0,50	0,35
105	2017.01	0	39	1,39	0,36	0,72	0,72	1,00	1,68	0,62	0,52	0,00	0,24
106	2017.01	0	21	0,67	0,51	0,52	0,63	0,61	1,58	0,30	0,40	0,74	0,95
107	2017.01	0	54	0,65	0,54	0,50	0,42	0,96	1,12	0,39	0,29	0,00	0,59
108	2017.01	1	36	1,13	0,70	0,57	0,60	1,18	1,98	1,06	1,19	1,27	0,74
109	2017.01	1	37	0,88	0,89	0,83	0,57	0,90	0,92	0,74	0,95	0,73	0,79
110	2017.01	0	37	1,40	0,70	0,91	0,52	0,76	1,22	0,82	0,73	1,01	1,15
111	2017.01	0	72	1,06	0,66	0,56	0,57	0,66	1,38	0,48	0,56	1,13	1,42
112	2017.02	0	23	1,16	0,48	0,79	0,83	0,90	1,22	0,72	0,69	0,67	0,96
113	2017.02	1	37	0,90	0,85	0,82	0,87	0,40	1,08	1,16	0,53	0,45	0,63
114	2017.02	0	49	0,99	0,62	0,92	0,75	0,89	1,28	1,04	0,80	1,26	1,28
115	2017.03	0	40	1,42	0,73	0,63	0,48	0,50	0,74	0,71	0,53	1,12	1,26
116	2017.03	0	38	1,04	0,39	0,36	0,50	0,58	0,84	0,90	0,15	0,48	0,37
117	2017.03	0	56	1,21	0,41	0,44	0,70	0,89	0,69	0,47	0,54	0,56	0,42
118	2017.03	0	25	1,43	1,56	0,65	0,52	0,54	0,69	0,34	0,53	0,21	0,26
119	2017.04	0	40	1,61	0,66	0,43	0,61	0,66	0,93	0,21	0,28	0,68	0,92
120	2017.04	0	32	1,23	0,55	0,81	0,68	0,67	0,82	0,68	0,78	1,10	1,10
121	2017.04	1	36	1,23	0,71	0,55	0,72	0,78	0,86	0,72	0,79	0,89	0,60
122	2017.04	0	62	1,31	0,96	0,73	0,65	0,70	0,59	1,08	0,95	1,06	1,09
123	2017.05	0	28	0,89	0,84	0,88	0,59	0,59	1,14	0,83	0,97	0,70	0,80
124	2017.05	0	34	1,77	1,36	0,72	0,51	0,59	0,49	0,38	0,38	1,00	0,34
125	2017.05	0	34	1,66	0,58	0,72	0,82	0,64	1,26	0,45	0,46	1,31	1,31
126	2017.05	0	42	1,62	0,56	0,60	0,54	0,98	1,04	0,42	0,51	1,05	1,20
127	2017.05	1	48	1,10	0,54	0,58	0,45	0,64	1,05	0,84	1,08	0,88	1,12
128	2017.05	0	53	1,01	0,35	0,58	0,39	0,65	0,74	0,87	0,78	0,77	0,86
129	2017.06	1	34	0,96	0,51	0,58	0,33	0,50	1,16	0,63	0,83	0,56	0,86
130	2017.06	0	27	0,96	0,58	0,61	0,48	0,36	0,99	0,90	0,82	0,45	0,34
131	2017.06	1	68	1,23	0,72	0,71	0,69	0,49	1,09	0,49	0,70	0,58	0,89
132	2017.06	1	68	2,00	1,29	0,86	0,59	0,78	1,13	0,82	0,84	0,83	0,66
133	2017.06	1	19	1,59	0,85	0,52	0,29	0,58	1,05	0,74	0,67	0,74	0,87
134	2017.06	0	22	0,99	0,52	0,53	0,41	0,55	0,74	0,45	0,74	0,69	0,80
135	2017.06	1	35	1,15	0,63	0,61	0,34	0,46	0,84	0,35	0,54	0,76	0,74
136	2017.06	0	36	1,13	0,49	0,53	0,65	0,42	0,94	0,82	0,58	0,99	0,51
137	2017.06	0	40	1,16	0,65	0,59	0,69	0,72	0,87	0,34	0,46	0,64	0,98
138	2017.06	0	62	0,93	0,50	0,56	0,59	0,53	0,63	0,62	0,29	0,54	0,48
139	2017.06	0	21	1,24	0,62	0,67	0,39	0,46	0,55	0,57	0,32	0,77	0,55
140	2017.06	1	24	1,44	0,78	0,49	0,65	0,61	0,94	0,69	0,85	0,54	0,72
141	2017.06	0	25	0,98	0,43	0,58	0,55	0,65	0,81	0,50	0,29	0,63	0,63
142	2017.07	1	19	0,97	0,43	0,33	0,84	0,68	0,38	0,99	1,20	0,77	1,07

143	2017.07	1	26	0,96	0,76	0,53	0,52	0,59	0,79	1,03	0,69	1,10	1,10
144	2017.08	1	23	1,15	1,13	0,35	0,80	0,46	0,81	0,94	1,12	0,75	0,46
145	2017.08	0	34	0,85	0,37	0,55	0,44	0,34	0,69	0,49	0,57	0,67	0,78
146	2017.08	1	17	1,58	0,64	0,69	0,65	0,69	0,78	0,62	0,67	0,62	0,62
147	2017.08	0	25	1,26	0,60	0,53	0,55	0,80	1,20	0,56	0,51	0,84	1,04
148	2017.09	1	54	0,95	0,78	0,65	0,99	1,14	1,04	0,41	0,36	0,71	0,62
149	2017.09	1	78	2,09	0,67	0,58	0,76	0,74	1,20	0,54	0,33	1,22	1,27
150	2017.09	1	22	1,90	0,72	0,76	0,67	0,76	0,94	0,71	0,61	0,77	0,53
151	2017.09	1	82	1,34	0,87	0,42	0,68	0,53	0,92	0,43	0,68	0,96	0,54
152	2017.09	0	59	1,30	0,79	0,71	0,64	0,47	1,06	0,55	0,79	0,65	0,92
153	2017.09	1	61	0,65	0,56	0,72	0,71	0,57	0,69	0,56	0,83	0,72	0,89
154	2017.09	0	38	0,91	0,44	0,54	0,49	0,60	0,70	0,49	0,50	0,46	0,40
155	2017.09	0	25	1,09	0,48	0,24	0,31	0,30	0,79	0,92	0,92	0,69	0,62
156	2017.09	1	30	1,31	0,46	0,36	0,46	0,44	0,89	0,69	0,72	0,85	1,32
157	2017.10	1	58	0,73	0,25	0,54	0,78	0,87	0,82	0,28	0,21	0,62	0,76
158	2017.10	0	64	0,96	0,32	0,90	0,75	0,98	0,89	0,91	0,66	0,31	0,69
159	2017.10	1	38	0,38	0,32	0,86	0,76	0,49	1,09	0,64	0,72	1,06	1,20
160	2017.11	0	39	0,57	1,10	0,65	0,53	0,53	1,13	0,37	0,14	0,56	0,41
161	2017.12	1	59	1,02	0,73	0,48	0,75	0,77	0,92	0,37	0,59	0,63	1,12
162	2017.12	1	36	0,71	0,55	0,37	0,36	0,62	1,21	0,76	1,50	0,96	1,22
163	2017.12	1	41	1,20	0,95	0,61	0,53	0,63	1,05	0,73	0,49	0,70	0,57
164	2018.01	1	50	1,32	0,94	0,64	0,72	0,71	0,62	1,09	1,04	1,09	1,09
165	2018.01	1	69	1,41	0,64	0,84	0,93	0,78	1,41	1,07	1,12	1,14	0,99
166	2018.01	0	23	1,47	0,80	0,55	0,42	0,56	0,85	0,37	0,48	0,93	1,01
167	2018.01	1	35	1,33	0,47	0,43	0,31	0,57	0,91	0,93	0,43	0,68	1,15
168	2018.01	0	62	0,89	0,78	0,56	0,59	0,51	0,92	0,64	0,69	0,84	0,54
169	2018.01	0	81	0,85	0,59	0,55	0,64	0,93	0,70	1,08	0,99	0,95	0,81
170	2018.01	0	55	0,94	0,43	0,36	0,24	0,54	1,24	0,81	0,77	0,85	1,12
171	2018.01	1	59	1,24	0,46	0,36	0,46	0,61	1,19	0,81	0,99	0,88	1,19
172	2018.01	1	41	1,16	0,47	0,39	0,62	0,83	0,86	0,69	0,56	0,38	0,76
173	2018.01	0	41	0,93	0,64	0,68	0,45	0,33	0,34	0,69	0,53	0,76	0,90
174	2018.02	0	37	1,05	0,87	0,34	0,35	0,51	0,23	0,55	0,72	0,73	0,60
175	2018.02	0	41	0,76	0,57	0,54	0,43	0,53	0,36	0,71	0,71	0,63	0,68
176	2018.02	0	44	1,46	0,45	0,44	0,68	0,41	0,97	0,51	0,34	0,84	0,86
177	2018.02	1	79	1,11	0,58	0,61	0,70	0,59	0,30	0,74	0,69	0,92	0,95
178	2018.02	1	67	1,04	0,86	0,74	0,74	0,57	0,88	0,94	0,86	0,95	0,91
179	2018.02	0	12	1,14	0,60	0,45	0,51	0,43	0,58	0,19	0,37	0,58	0,58
180	2018.03	1	72	1,03	1,01	0,43	0,51	1,24	1,07	0,78	0,61	0,89	0,50
181	2018.03	1	41	1,12	0,65	0,58	0,53	0,76	1,29	0,86	0,86	0,65	0,66
182	2018.03	0	18	1,08	0,51	0,37	0,35	0,24	0,89	0,44	0,39	0,17	0,62
183	2018.03	0	59	1,03	0,47	0,34	0,36	0,33	0,26	0,63	0,40	0,90	0,73
184	2018.04	1	20	1,34	0,44	0,38	0,44	0,41	0,81	0,59	0,51	1,15	1,08
185	2018.04	0	60	1,29	0,59	0,65	0,97	0,92	0,82	0,33	0,54	0,18	0,18
186	2018.04	0	30	1,23	0,98	0,60	0,53	0,58	0,70	0,57	0,52	0,51	0,75
187	2018.04	0	57	0,95	0,63	0,53	0,84	0,98	1,37	0,97	0,88	0,73	0,73
188	2018.04	1	62	1,40	0,62	0,58	0,96	0,86	0,88	0,82	0,58	0,45	0,32
189	2018.04	1	70	1,14	0,64	0,69	0,45	0,71	0,72	0,82	1,46	0,41	0,79
190	2018.05	0	36	1,39	0,94	0,81	0,71	0,70	0,63	1,12	0,91	1,01	1,05
191	2018.05	1	40	0,69	0,61	0,46	0,43	0,44	1,23	1,32	1,30	1,06	1,08
192	2018.05	0	22	1,43	0,57	0,39	0,24	0,28	1,14	0,59	0,57	0,86	0,80
193	2018.05	0	52	1,48	0,71	0,67	0,50	0,70	0,74	0,73	0,28	0,69	0,89
194	2018.05	1	49	1,12	0,53	0,69	0,46	0,63	0,81	0,69	0,54	0,75	0,85
195	2018.05	1	41	1,07	0,72	0,46	0,55	0,92	1,09	1,24	1,14	1,02	1,26

196	2018.05	0	51	0,79	0,29	0,30	0,46	0,32	0,89	1,06	0,75	0,75	0,69
197	2018.05	1	81	1,37	0,62	0,56	0,39	0,72	0,56	0,59	0,63	0,74	0,26
198	2018.05	1	39	1,43	0,42	0,55	0,47	0,50	1,09	0,55	0,78	1,01	0,99
199	2018.06	0	51	0,76	0,24	0,40	0,62	0,34	0,92	1,41	1,13	0,86	0,88
200	2018.06	0	32	0,94	0,78	0,72	0,57	0,36	0,72	0,64	0,66	1,07	0,89

2								3								
C4 R	C4 L	C5 R	C5 L	C6 R	C6 L	C7 R	C7 L	C2 R	C2 L	C3 R	C3 L	C4 R	C4 L	C5 R	C5 L	C6 R
0,29	0,14	1,00	0,67	1,46	1,96	1,93	2,04	13	14	21	16	21	3	16	15	13
0,94	0,69	1,01	0,75	0,84	0,82	2,85	2,96	7	18	20	17	22	13	17	13	15
0,90	0,99	1,17	1,37	1,42	1,35	2,20	2,21	12	12	9	0	20	18	13	14	15
0,99	1,11	1,06	1,05	0,27	0,49	0,56	0,59	17	13	22	9	20	24	10	18	26
0,78	0,81	1,29	1,15	1,58	1,28	2,02	1,64	5	8	23	12	23	10	23	21	16
0,64	0,71	0,64	0,69	1,16	1,27	1,59	1,67	29	5	10	10	21	16	15	13	6
0,28	0,64	0,42	0,60	0,30	0,50	2,00	2,24	15	17	12	15	28	23	14	23	15
0,75	1,70	0,70	1,10	1,44	1,00	2,25	2,77	17	8	14	12	7	6	26	10	6
0,62	0,59	0,64	0,89	0,75	0,74	1,53	1,61	25	11	14	7	11	6	14	9	19
0,97	1,12	0,84	1,03	1,16	0,79	1,08	1,44	13	17	9	14	5	8	6	13	8
0,51	0,74	0,76	0,72	0,84	0,52	1,18	1,18	16	3	5	6	7	13	22	19	20
1,20	0,38	0,87	0,68	1,70	1,07	2,95	2,96	14	22	6	17	7	19	7	14	14
0,86	0,85	0,95	1,11	1,54	1,12	1,51	1,96	14	12	16	5	24	14	14	19	18
0,75	0,66	0,73	0,90	1,15	1,44	1,93	2,14	26	4	5	13	12	8	20	13	7
0,98	0,57	0,79	0,86	1,11	1,04	1,79	1,90	24	19	22	25	21	19	24	29	24
0,39	0,60	0,76	0,77	1,04	1,06	0,93	1,51	14	19	15	21	6	6	6	7	23
0,87	0,68	0,69	0,93	1,57	1,73	2,40	2,30	6	11	12	17	19	13	17	22	12
0,62	0,57	0,60	0,66	1,13	1,03	1,95	2,06	22	18	21	16	19	14	17	22	21
0,74	0,78	0,82	0,69	0,86	0,96	1,38	1,46	6	12	13	21	23	26	28	23	16
0,67	0,56	0,80	0,67	0,47	0,63	1,05	1,09	12	19	2	5	7	10	12	13	15
0,53	0,53	0,86	0,94	1,02	1,35	1,13	1,16	15	13	18	14	12	7	7	15	8
0,62	0,67	0,78	0,53	1,30	1,67	2,78	2,16	9	7	26	20	16	15	11	25	16
0,76	0,50	1,07	1,21	0,61	0,74	1,29	1,35	15	12	15	15	13	7	15	13	15
0,72	1,19	1,16	1,88	1,81	2,06	1,89	19,00	16	15	10	21	19	14	14	10	9
0,72	0,44	0,85	0,74	1,27	1,36	2,36	2,21	12	13	-23	23	27	22	12	18	13
1,09	0,67	0,87	0,87	1,32	0,90	1,67	1,31	19	18	5	3	3	0	14	10	16
0,95	0,99	0,98	0,79	1,52	1,38	1,81	1,53	14	15	26	17	27	16	25	15	14
0,83	0,62	0,74	0,86	1,27	1,21	1,68	0,99	16	7	5	12	7	7	8	3	16
0,60	0,60	0,85	0,36	0,96	0,84	1,97	2,45	13	8	11	11	12	12	9	8	6
1,17	0,87	0,18	0,34	0,35	1,13	1,24	1,76	12	5	21	19	9	12	9	23	10
0,49	0,73	0,64	0,97	0,98	0,60	1,34	1,50	15	15	5	9	13	16	7	10	21
0,95	1,13	0,93	0,71	0,86	0,96	1,05	1,20	18	24	23	21	20	15	16	12	14
0,48	0,83	0,74	1,00	1,21	1,42	1,46	1,68	17	25	22	16	10	14	17	17	18
1,36	1,54	0,71	0,25	0,26	0,53	1,04	1,17	16	13	17	24	24	12	10	8	15
0,56	0,51	0,47	0,47	0,90	0,51	2,27	2,38	3	6	16	20	9	15	11	13	18
0,34	0,43	0,58	0,69	0,64	0,80	1,53	1,89	20	16	14	8	23	17	15	12	15

0,64	0,40	0,64	0,88	0,94	0,98	1,51	1,47	12	9	8	7	7	5	20	24	9
0,80	0,34	0,13	0,11	0,38	0,56	2,51	2,18	19	11	11	9	5	8	8	12	18
0,68	0,83	0,87	0,53	1,14	1,16	1,25	1,32	15	16	18	15	9	7	16	10	21
1,38	0,98	1,16	1,15	1,39	1,37	2,35	2,20	24	14	13	19	24	15	26	14	18
0,72	0,61	0,69	0,76	1,34	1,38	1,75	1,56	25	21	14	12	23	15	20	16	8
0,69	0,94	1,16	1,01	1,51	1,47	2,26	2,20	8	9	0	21	20	32	6	11	11
0,88	0,75	1,07	1,15	1,43	1,75	1,95	1,93	17	14	32	25	26	26	23	23	13
0,86	0,96	1,12	1,02	1,18	1,18	2,29	2,44	14	12	24	20	29	29	18	15	-1
0,63	1,09	1,09	1,09	1,22	1,49	2,00	2,00	6	15	19	14	25	17	16	17	6
0,85	0,85	0,95	1,13	1,05	1,08	2,08	2,09	23	7	21	28	20	18	25	27	14
1,26	1,04	1,10	1,20	1,48	1,45	1,77	1,83	22	15	18	17	32	26	23	22	20
0,72	0,83	0,92	1,41	1,41	0,99	1,85	1,80	17	17	17	24	16	18	7	14	5
0,78	1,06	1,08	1,30	2,15	2,12	2,61	2,49	8	18	16	18	13	38	0	25	6
1,25	1,59	1,46	1,57	1,60	1,65	2,13	2,02	14	15	11	18	20	12	17	11	14
0,96	1,27	1,73	1,63	2,33	2,23	2,54	2,62	13	12	-9	9	29	20	12	12	9
0,88	0,57	1,04	1,22	1,71	2,10	2,48	2,59	18	19	22	17	26	23	9	14	22
1,04	1,43	0,88	1,27	1,55	1,51	2,31	2,27	29	25	32	25	21	21	26	34	14
1,16	1,36	1,56	1,52	1,75	1,87	2,71	2,83	12	5	15	7	18	11	8	9	13
1,18	0,83	1,00	1,39	1,86	1,82	2,24	2,22	12	12	15	18	16	8	14	16	8
0,67	0,98	0,82	0,97	1,59	1,54	2,06	1,82	1	12	14	-14	16	15	13	12	11
0,75	0,76	1,18	0,93	1,44	1,54	2,12	2,07	20	26	8	19	17	30	4	23	10
0,35	0,58	0,85	0,85	0,97	0,95	1,38	1,56	20	12	23	18	19	2	0	0	24
0,97	1,09	1,16	1,14	1,50	1,57	1,88	1,88	20	21	15	23	20	31	13	16	8
0,86	1,06	0,83	1,20	1,20	1,42	1,81	1,79	18	18	25	26	16	18	9	12	11
0,45	0,58	0,67	0,70	1,17	1,36	1,76	1,96	13	9	17	15	8	19	10	18	15
0,78	0,91	1,23	1,07	1,68	1,85	1,97	1,95	8	7	3	-3	15	11	8	8	11
1,05	0,95	0,95	1,15	1,93	1,98	2,30	2,26	41	32	16	15	23	18	9	5	7
0,87	0,95	0,21	0,80	0,40	0,41	1,72	1,75	12	18	13	13	10	7	6	5	6
1,14	1,51	1,24	1,77	2,32	2,19	2,59	2,66	30	20	25	18	18	22	20	20	2
0,91	1,01	0,87	0,98	1,32	1,43	2,15	2,05	14	11	17	17	24	23	22	21	14
0,71	1,09	0,95	1,00	1,71	1,67	2,06	1,98	13	12	0	0	18	19	14	7	12
0,60	0,79	0,84	0,76	1,14	1,31	1,70	1,18	10	11	27	19	11	10	20	17	16
1,03	0,91	0,86	0,93	1,12	1,00	1,86	1,87	9	9	15	25	24	26	21	22	10
0,72	0,78	0,81	0,82	0,78	0,70	1,92	1,85	7	4	17	7	26	18	13	7	16
1,01	1,23	0,99	1,17	1,39	1,51	1,73	1,67	10	13	14	11	25	23	15	16	8
0,75	0,72	1,19	1,16	1,88	1,81	2,06	1,89	19	16	15	10	21	19	14	14	10
0,75	0,61	0,55	0,75	1,00	0,93	1,73	1,72	25	26	17	6	12	25	25	22	23
0,96	0,81	0,62	0,98	1,28	1,16	2,17	2,18	9	12	18	15	21	18	12	9	12
0,94	0,85	0,96	1,00	1,92	2,13	2,00	2,43	15	10	15	9	18	21	16	15	8
1,24	0,92	0,86	0,56	1,25	1,26	2,35	2,08	13	10	18	12	24	23	9	18	9
0,72	0,44	0,85	0,74	1,27	1,36	2,36	2,21	12	13	-23	23	27	22	12	18	13
1,00	1,00	1,03	0,97	1,40	1,41	1,65	1,65	20	18	29	31	20	21	19	21	19
0,82	0,94	0,64	0,64	1,03	1,03	1,68	1,57	11	11	10	13	24	24	6	6	3
0,90	1,08	0,92	1,19	1,45	1,36	1,75	1,67	12	3	14	25	24	25	19	21	13
0,48	1,04	0,72	0,71	1,18	0,77	1,65	1,50	10	12	14	25	20	26	12	23	7
0,65	0,88	0,50	0,58	0,82	0,86	1,78	1,64	11	14	14	17	10	17	11	18	8
0,54	0,57	0,59	0,63	0,48	0,33	0,98	1,28	20	22	26	17	15	19	12	16	12
0,95	1,16	0,93	0,78	1,00	0,89	1,58	1,58	10	16	16	15	26	22	19	15	11
0,60	0,79	0,78	0,79	1,02	1,03	1,68	1,68	12	10	13	16	21	26	20	20	11
1,08	0,97	1,19	1,21	1,66	1,49	1,72	1,69	14	12	5	13	9	13	8	11	8
0,74	0,66	1,00	1,39	1,67	1,67	2,08	1,99	15	18	19	12	10	5	30	26	10
0,66	1,16	0,98	0,90	1,23	1,44	1,90	2,00	14	19	10	15	8	22	13	15	10
1,33	0,73	1,15	0,90	1,05	1,09	2,03	1,96	19	12	27	24	22	11	24	27	8

0,00	0,77	1,21	1,02	1,76	1,73	1,50	2,15	12	21	19	27	-13	13	7	8	9
0,61	0,64	0,91	0,68	1,35	1,38	1,84	1,74	13	14	-8	36	25	28	32	17	12
1,06	0,91	0,82	0,85	1,01	0,83	1,60	1,51	10	14	9	30	7	26	19	16	13
0,00	0,91	1,02	0,65	1,15	1,10	1,17	1,26	16	16	1	-1	-21	21	22	24	18
1,31	1,10	1,50	1,29	2,00	1,70	2,60	2,37	18	19	4	20	26	-10	12	16	15
0,63	0,63	1,04	1,06	1,25	1,20	1,73	1,73	20	21	21	19	24	21	6	12	9
0,78	1,08	1,15	1,12	1,43	1,34	2,01	2,08	24	21	18	28	24	29	19	17	9
0,83	0,74	1,00	1,21	1,42	1,46	1,68	1,70	25	22	16	10	14	17	17	18	10
0,62	0,99	1,01	1,04	1,02	1,16	1,72	1,72	22	21	26	22	8	20	22	16	10
0,75	0,82	1,14	1,14	1,84	1,74	1,97	1,92	19	17	17	29	19	25	19	-6	10
0,67	1,12	0,70	1,11	1,63	1,74	1,53	1,53	22	28	17	25	26	28	5	12	17
1,61	1,58	1,28	1,61	1,70	1,87	2,83	2,75	11	13	13	14	20	24	19	19	12
0,78	1,03	1,08	0,87	1,53	1,56	1,80	1,79	10	11	19	19	15	13	0	13	5
0,46	0,50	0,71	0,72	1,57	1,48	1,33	1,32	10	11	21	31	20	23	5	15	4
0,81	0,91	0,82	0,78	1,13	1,24	2,14	2,12	20	27	20	18	31	22	28	31	6
0,87	0,73	1,02	1,14	1,20	1,13	1,32	1,27	18	16	-9	9	24	18	4	8	10
1,13	0,93	0,71	0,86	0,96	1,05	1,20	1,08	24	23	21	20	15	16	12	14	12
0,62	0,51	0,45	0,46	1,05	0,97	1,45	1,33	22	18	-21	21	26	18	22	18	18
0,87	0,79	1,29	0,84	0,97	1,17	1,46	1,51	10	13	18	13	10	35	7	10	8
0,48	0,52	1,04	1,11	1,54	1,58	2,14	2,18	12	14	20	28	26	23	13	21	9
0,82	0,57	0,28	0,87	1,09	0,34	1,91	1,86	31	25	29	27	35	23	13	21	20
0,93	1,05	0,82	1,00	1,02	1,15	1,73	1,78	13	10	27	18	32	34	22	26	15
0,82	0,77	0,68	0,65	1,29	1,21	1,73	1,51	20	15	18	23	32	31	19	28	12
0,91	0,92	0,59	0,86	1,75	1,82	1,48	1,56	9	11	18	18	26	8	18	11	19
1,08	1,08	0,96	0,56	1,12	1,23	1,74	1,63	17	14	14	16	13	16	13	2	5
0,81	1,25	0,98	1,36	1,26	1,39	2,39	2,41	10	15	15	8	14	15	19	21	28
0,63	0,17	0,98	1,17	1,29	1,23	1,94	1,92	13	14	8	18	17	15	9	8	14
1,01	0,49	0,51	0,68	0,57	0,63	0,58	0,76	2	2	18	16	15	12	6	17	20
0,46	0,64	0,73	0,99	1,03	2,01	1,73	1,56	6	4	13	17	15	10	14	13	13
1,07	0,99	0,92	1,14	1,17	1,01	0,48	0,52	5	8	13	11	10	14	15	16	13
0,47	0,47	0,73	0,72	1,14	1,48	2,17	2,37	3	1	13	13	2	2	10	11	9
0,79	0,47	0,41	0,37	1,26	1,17	2,66	2,83	5	7	13	6	12	6	20	4	7
1,16	1,23	1,76	1,68	1,98	2,01	2,43	2,55	7	7	9	6	15	12	19	15	15
0,51	0,50	0,76	0,86	1,43	1,55	1,85	1,89	3	7	7	4	9	13	8	8	14
0,89	0,75	0,95	1,06	1,13	1,20	1,39	1,47	13	5	6	5	5	5	23	14	11
0,87	0,74	0,80	0,83	1,09	1,19	2,13	2,37	12	14	19	19	7	8	5	10	15
0,81	0,96	0,92	0,92	1,79	1,73	2,40	2,38	7	8	16	14	10	12	15	18	13
0,80	0,82	1,49	1,57	1,70	1,76	2,31	2,17	10	5	24	20	14	21	25	19	10
0,69	0,69	0,99	0,46	1,63	1,67	2,04	2,04	5	8	25	18	15	15	27	25	19
0,58	0,49	1,10	0,99	1,41	1,45	2,25	2,41	10	12	22	8	10	8	17	18	15
0,94	0,51	0,67	0,76	0,92	1,13	1,78	1,83	6	6	17	6	19	11	9	14	13
0,84	0,99	1,23	1,01	1,24	1,46	2,00	1,98	8	3	12	8	7	8	13	6	21
0,89	0,66	1,04	0,99	1,22	1,14	2,53	2,57	4	3	4	4	12	8	11	6	9
0,87	1,03	1,03	1,01	1,96	1,95	2,76	2,62	9	5	6	7	9	14	26	18	22
0,29	0,47	0,25	0,43	0,63	0,67	1,60	1,78	5	2	12	17	7	5	7	9	17
0,69	0,74	0,92	0,92	1,64	1,79	2,24	2,27	7	7	13	6	14	9	9	15	16
0,41	0,35	0,24	0,27	1,12	0,71	1,90	1,83	22	4	21	7	15	15	11	17	19
0,59	0,59	0,73	0,65	0,98	1,14	1,98	2,00	4	8	15	11	14	10	19	18	13
0,45	0,69	0,27	0,42	0,49	0,42	0,95	1,22	16	13	6	11	5	4	16	8	8
0,69	0,79	0,61	0,76	0,95	1,17	1,08	0,92	5	3	12	8	8	5	12	22	18
0,75	0,82	0,77	0,89	1,79	1,29	1,77	1,83	4	7	11	7	11	8	13	16	14
0,18	0,32	0,55	0,45	0,91	0,81	1,89	1,89	7	27	19	15	9	16	11	6	15
0,51	0,75	1,51	1,48	2,07	2,17	2,29	2,65	12	8	14	18	26	18	7	10	25

0,87	0,39	1,02	1,10	1,05	1,16	1,88	1,51	13	8	18	18	17	10	9	7	13
0,63	0,75	1,15	1,15	1,48	1,44	2,91	2,79	7	9	8	15	12	17	13	10	19
0,18	0,18	0,73	0,91	0,59	0,42	1,34	1,40	5	6	18	22	7	7	10	11	15
0,73	0,86	0,53	0,74	1,02	1,19	1,67	0,98	8	5	19	13	15	9	16	14	15
0,69	0,84	0,99	0,94	1,70	1,74	1,71	1,66	8	3	26	10	13	14	21	14	21
0,87	0,96	0,81	0,92	1,01	1,04	0,52	0,47	4	11	12	9	11	21	16	19	13
0,91	1,14	0,82	0,91	1,64	1,75	3,02	2,49	6	7	17	22	16	25	14	16	26
1,01	0,54	1,13	0,77	0,54	0,49	1,45	1,38	11	14	10	12	14	8	7	12	23
0,53	0,87	1,10	0,96	1,53	1,43	1,99	2,05	8	7	12	16	18	13	16	12	19
0,82	0,97	1,19	1,00	1,44	1,39	1,93	1,96	7	2	10	8	5	8	17	8	23
0,35	0,42	1,05	0,74	0,85	1,19	1,65	1,95	12	6	15	16	9	12	13	17	11
0,28	0,28	0,40	0,70	1,11	0,99	1,92	1,81	7	15	22	11	10	10	8	13	20
0,82	0,67	0,85	0,86	1,35	1,11	2,64	2,55	17	24	12	9	23	20	15	17	24
0,73	1,14	0,93	0,69	1,46	1,45	1,34	1,21	7	8	25	17	23	25	16	9	25
1,03	0,98	0,75	1,02	0,95	1,02	0,52	0,39	6	10	13	10	9	17	14	11	13
0,60	0,41	0,70	0,82	2,07	2,05	2,62	2,48	8	5	19	25	3	6	7	10	22
0,42	0,51	0,68	0,69	1,12	1,26	1,60	1,10	3	3	15	12	7	8	10	12	7
0,76	1,00	0,77	0,83	1,15	0,66	2,13	2,09	21	28	13	12	17	8	17	14	29
0,65	0,53	0,48	0,42	1,38	1,24	2,43	2,49	2	8	12	10	10	16	21	11	7
1,22	1,29	1,09	1,31	1,15	1,34	1,77	1,65	18	11	25	12	27	22	10	7	17
0,88	1,16	1,27	0,97	1,30	1,28	2,19	2,20	7	3	15	11	6	12	14	10	10
1,18	1,26	1,84	1,73	1,90	2,01	2,23	2,46	9	9	9	7	15	14	17	14	16
0,92	0,85	1,05	1,09	1,25	1,39	1,61	1,90	9	7	10	10	6	4	7	5	5
1,03	0,69	0,95	1,07	1,08	1,15	1,71	1,87	6	3	9	5	7	11	12	13	7
0,74	0,76	0,64	1,15	1,92	1,91	2,90	2,87	5	4	13	8	13	17	15	14	14
0,44	0,58	0,58	0,74	0,99	1,06	2,09	2,15	12	13	4	7	10	9	18	8	12
0,90	0,81	0,94	1,11	1,96	1,97	2,41	2,39	15	18	11	15	7	21	7	5	8
0,77	0,84	1,32	1,13	1,69	1,66	2,24	2,21	13	10	12	16	17	21	20	19	12
0,91	0,42	0,79	0,74	1,70	1,49	2,35	2,52	10	6	5	14	14	12	14	12	8
0,99	0,94	0,92	0,90	0,73	1,02	2,39	2,43	7	8	15	12	16	11	9	11	7
0,99	0,81	0,89	0,94	1,01	1,03	1,59	1,63	7	5	12	8	10	9	17	13	21
0,93	0,99	1,19	1,17	1,45	1,68	2,01	2,05	6	5	9	6	17	16	18	9	13
0,61	0,45	0,54	0,46	1,24	0,98	2,32	2,37	9	8	12	14	6	8	8	13	14
0,59	0,64	0,70	0,74	1,34	1,47	1,85	1,84	5	7	14	6	10	6	11	10	16
0,79	0,79	1,16	0,60	1,24	1,07	1,64	1,68	6	6	21	10	14	14	6	7	12
1,05	1,26	1,33	1,40	2,01	2,02	2,68	2,74	7	12	6	7	6	8	7	5	25
0,72	0,40	0,89	0,41	1,28	1,39	1,51	1,48	4	13	6	6	13	15	14	7	12
0,90	0,79	0,92	1,05	1,21	0,66	1,93	1,92	6	4	8	4	12	14	7	11	4
0,80	0,89	1,30	1,23	1,71	1,78	2,23	2,25	8	8	9	5	6	3	10	6	15
0,62	0,64	0,77	0,75	1,14	1,14	1,90	1,81	9	4	11	5	11	7	8	11	17
0,89	0,84	0,93	0,97	1,33	1,23	1,92	1,98	9	4	10	12	12	13	19	15	20
1,15	1,01	0,71	1,01	0,48	0,88	2,24	2,25	4	5	11	10	15	9	10	10	8
0,60	0,12	0,50	0,93	0,67	0,89	1,72	1,81	8	4	8	8	11	13	5	7	4
0,82	0,69	0,84	0,95	0,92	1,18	1,74	1,71	10	8	11	15	10	15	17	21	14
1,33	1,00	1,15	1,26	1,79	1,90	2,11	2,34	14	10	11	11	11	9	7	6	11
0,99	0,94	0,80	0,51	0,85	0,91	1,77	1,76	9	5	9	6	14	12	10	6	10
0,83	0,74	1,70	1,81	2,01	2,03	2,97	2,75	14	10	17	9	10	7	15	13	9
1,16	1,26	1,34	1,43	1,91	1,99	2,31	2,35	9	11	10	6	13	11	18	12	13
1,19	0,73	0,89	0,98	1,43	1,37	2,46	2,61	18	20	10	6	14	16	13	15	20
0,98	1,03	0,94	1,02	1,07	1,19	1,68	1,67	7	4	10	5	11	12	18	20	13
0,90	0,81	1,03	1,04	1,11	1,26	2,20	2,29	11	5	6	8	8	7	10	16	10
0,82	0,52	0,90	1,17	1,17	1,27	2,35	2,45	8	7	10	9	10	4	11	10	8
1,47	1,24	1,37	1,54	2,04	2,22	2,73	2,76	5	7	12	13	16	14	11	15	12

0,94	0,80	0,96	1,49	1,74	1,74	2,08	2,20	15	8	17	22	11	10	11	13	21
0,26	0,61	0,90	0,68	0,87	1,34	1,28	1,89	5	13	21	8	9	10	12	15	23
1,05	1,09	1,41	1,12	1,62	1,73	2,40	2,56	3	6	10	14	19	13	21	16	16
1,47	1,47	0,97	1,54	1,48	1,17	2,20	2,20	11	7	15	14	16	16	13	11	15
0,97	1,03	1,05	1,07	1,03	1,05	1,83	1,88	7	5	11	7	6	9	9	12	15

			4													
C6 L	C7 R	C7 L	C2 R	C2 L	C3 R	C3 L	C4 R	C4 L	C5 R	C5 L	C6 R	C6 L	C7 R	C7 L	C2 R	C2 L
12	14	3	147	56	63	130	279	614	58	122	72	33	15	7	0,16	0,24
10	14	24	118	20	45	34	59	93	59	131	110	134	33	23	0,25	0,17
17	13	18	102	119	58	124	69	94	91	58	45	31	41	45	0,46	0,57
8	6	9	122	77	31	60	45	68	91	83	552	300	177	188	0,31	0,27
16	20	18	164	92	66	39	156	181	34	51	50	64	39	49	0,30	0,17
12	9	11	229	147	155	100	128	125	75	52	33	30	40	43	0,53	0,23
19	13	16	135	198	214	296	214	155	124	82	117	46	14	29	0,37	0,26
2	25	8	198	82	95	36	115	65	89	51	51	125	33	61	0,36	0,43
13	22	19	138	126	92	74	74	103	108	64	103	109	41	37	0,36	0,48
11	9	7	153	433	655	64	76	81	56	20	9	129	90	78	0,46	0,52
20	15	12	865	114	80	46	92	95	26	79	85	154	43	63	0,37	0,63
8	16	7	177	114	94	174	58	211	55	72	30	39	41	13	0,33	0,23
11	5	7	142	181	295	248	69	186	65	53	13	26	57	43	0,22	0,27
4	8	13	197	233	81	114	109	215	101	40	56	30	39	31	0,58	0,47
20	11	14	144	108	55	83	40	156	75	57	85	61	55	30	0,59	0,59
8	14	9	176	82	198	106	167	237	54	108	40	65	42	40	0,43	0,39
17	9	8	144	600	343	79	97	171	62	13	15	60	36	30	0,49	0,52
22	17	13	145	154	88	59	103	125	107	76	75	94	32	28	0,60	0,42
21	18	12	202	77	233	104	81	97	54	87	21	48	54	53	0,24	0,20
13	11	13	269	314	165	255	113	114	45	52	98	60	64	51	0,19	0,23
11	7	14	150	142	119	67	98	215	44	48	62	50	47	46	0,34	0,38
12	18	12	123	85	59	72	94	152	127	102	69	60	22	31	0,55	0,34
10	20	18	154	108	90	104	82	134	64	55	102	62	59	37	0,38	0,29
6	5	173	85	107	65	133	236	47	73	44	55	36	38	6	0,37	0,28
13	8	6	127	75	0	66	100	245	79	115	0	74	31	20	0,30	0,37
9	12	7	110	156	40	63	53	181	60	31	79	69	65	51	0,31	0,28
15	24	12	117	75	47	41	73	85	77	67	64	75	52	65	0,29	0,45
14	4	7	126	263	85	86	49	160	47	28	53	65	49	63	0,22	0,16
8	8	15	116	67	110	149	140	175	91	242	51	58	30	24	0,32	0,25
10	17	24	182	132	71	61	32	118	372	112	266	67	94	49	0,43	0,46
19	14	14	133	276	178	80	94	111	81	34	37	143	37	49	0,40	0,41
12	15	7	6	223	128	70	66	54	170	42	47	77	90	94	0,14	0,97
10	10	7	29	510	139	92	110	100	188	20	38	51	33	49	0,82	0,20
10	12	19	94	114	49	74	53	58	111	196	346	162	131	132	0,42	0,48
23	16	9	500	131	114	55	107	106	60	62	40	139	25	21	0,55	0,55
7	19	22	183	73	69	61	174	140	60	123	105	115	22	23	0,60	0,74

9	12	10	163	131	111	79	102	185	77	58	69	58	42	27	0,42	0,44
12	13	16	80	93	264	141	103	453	700	927	66	66	32	16	0,42	0,53
16	9	10	169	126	68	69	99	143	45	100	68	58	54	63	0,23	0,25
14	9	7	106	116	55	55	38	110	101	83	92	98	59	45	0,47	0,63
8	5	8	206	73	92	269	160	297	135	128	53	25	41	39	0,59	0,62
7	7	6	177	119	102	262	129	139	57	69	44	46	31	43	0,39	0,33
14	10	8	145	49	97	98	93	173	87	85	45	52	45	39	0,49	0,51
1	6	5	226	88	79	26	84	140	51	57	53	76	38	39	0,36	0,38
16	5	7	147	119	115	93	149	129	74	59	50	61	32	55	0,53	0,48
12	6	9	101	101	75	78	84	169	135	61	96	87	41	41	0,48	0,30
17	10	11	251	160	68	74	71	133	63	36	68	63	71	57	0,41	0,47
14	8	8	149	68	80	159	64	76	83	65	54	34	39	46	0,41	0,44
9	7	7	127	58	42	63	87	95	93	59	55	39	30	43	0,20	0,33
8	7	6	76	147	49	43	66	92	62	24	98	85	59	79	0,41	0,59
8	8	6	82	57	0	72	86	97	73	60	0	41	38	48	0,45	0,67
8	14	8	94	172	143	96	122	200	121	38	37	42	35	22	0,53	0,55
14	8	9	224	119	100	155	115	121	81	67	65	37	45	63	0,80	0,78
13	7	6	99	107	112	66	85	104	80	76	58	64	43	48	0,42	0,48
7	4	5	77	83	103	53	64	173	101	71	43	75	53	37	0,58	0,37
5	9	4	112	63	70	124	119	103	110	93	51	29	33	54	0,32	0,39
15	8	8	154	70	94	143	104	89	55	115	58	27	35	37	0,63	0,61
23	22	18	177	57	80	72	91	90	31	76	84	95	25	37	0,63	0,38
10	9	6	107	56	53	33	64	84	76	97	80	72	52	58	0,37	0,43
13	8	7	188	103	81	77	59	122	80	54	65	61	48	59	0,57	0,56
8	11	8	172	68	148	64	138	157	97	114	38	72	26	30	0,44	0,32
9	9	7	122	150	111	0	123	153	63	50	39	0	40	47	0,45	0,46
8	7	6	290	126	76	60	58	111	64	64	63	57	46	42	0,69	0,62
14	11	13	332	237	98	156	107	67	148	58	205	200	51	54	0,60	0,63
11	3	4	83	65	60	65	102	99	106	76	50	55	44	57	0,54	0,48
12	6	5	169	136	90	55	109	97	71	48	64	80	42	49	0,33	0,32
9	10	4	85	61	159	193	85	64	84	69	17	17	34	55	0,22	0,34
20	7	12	120	72	85	80	132	182	98	142	72	70	35	67	0,42	0,35
11	7	7	175	182	137	115	104	147	64	61	81	104	55	49	0,40	0,33
11	21	13	291	200	135	113	96	114	68	45	71	91	38	42	0,26	0,30
7	9	7	108	62	48	57	56	107	107	70	73	58	58	74	0,43	0,33
9	6	5	173	85	107	65	133	236	47	73	44	55	36	38	0,49	0,37
17	13	9	150	404	125	114	92	167	87	35	72	45	43	35	0,52	0,21
9	9	9	158	185	100	96	85	141	106	53	66	78	44	37	0,44	0,38
11	6	10	123	113	72	107	86	165	95	70	46	41	47	35	0,47	0,56
7	7	2	100	63	12	21	60	136	155	161	327	306	53	44	0,49	0,51
13	8	6	127	75	0	66	100	245	79	115	0	74	31	20	0,30	0,37
20	10	10	136	113	112	70	90	194	92	73	69	68	61	61	0,48	0,52
3	0	13	305	370	94	177	120	165	31	31	84	58	49	60	0,34	0,44
12	9	8	94	156	86	87	83	156	112	53	52	63	51	65	0,45	0,39
22	9	8	63	91	62	52	190	158	124	80	76	148	29	69	0,41	0,27
7	10	4	180	79	53	113	135	169	152	131	141	113	37	54	0,44	0,34
12	6	7	152	84	104	121	137	179	107	87	160	161	55	45	0,29	0,55
10	13	6	149	108	76	107	118	101	82	92	85	98	60	73	0,38	0,34
11	9	10	91	90	81	71	135	149	101	106	75	78	36	47	0,37	0,35
7	7	6	125	74	89	53	91	139	92	92	43	68	63	57	0,49	0,40
7	7	7	126	63	95	65	116	195	94	73	49	59	36	33	0,28	0,27
7	11	2	101	131	67	82	142	97	90	61	82	76	35	58	0,33	0,38
21	9	11	108	43	11	8	61	148	89	141	589	858	66	37	0,51	0,51

9	4	11	179	59	47	43	0	108	50	81	49	44	0	36	0,51	0,42
8	4	10	101	81	146	54	103	180	82	107	30	57	33	37	0,25	0,32
13	8	10	450	107	53	103	45	154	20	65	96	70	66	60	0,24	0,37
13	11	10	95	58	140	0	0	149	100	163	37	0	0	72	0,50	0,44
11	10	3	55	48	60	70	95	121	107	122	58	68	50	46	0,45	0,50
10	9	6	112	78	110	121	129	183	86	75	50	48	36	36	0,49	0,42
8	9	5	239	61	65	63	83	88	63	63	55	72	39	52	0,53	0,44
10	7	7	510	139	92	110	100	188	20	38	51	33	49	44	0,20	0,42
18	5	7	160	100	80	72	152	124	80	72	81	83	36	58	0,50	0,47
9	9	8	75	41	87	84	95	115	104	101	46	58	38	43	0,30	0,52
-1	6	6	53	87	89	125	99	128	207	110	55	40	44	73	0,46	0,56
17	6	6	106	81	48	53	82	168	85	75	92	90	57	57	0,45	0,45
13	5	12	116	67	87	152	87	127	88	116	50	40	43	58	0,25	0,38
10	7	4	136	108	89	102	172	164	104	104	53	38	35	38	0,40	0,34
11	7	8	209	107	118	174	72	116	80	95	44	28	38	43	0,54	0,43
10	9	10	224	69	0	300	115	230	61	46	0	21	66	57	0,47	0,44
15	7	9	223	128	70	66	54	170	42	47	77	90	94	86	0,97	0,69
6	13	8	167	186	0	71	155	220	87	63	0	61	43	38	0,65	0,54
11	7	9	107	59	45	81	136	251	82	142	131	63	60	52	0,36	0,43
10	7	7	119	94	114	72	188	177	71	86	47	50	22	24	0,44	0,36
12	9	5	171	96	90	45	93	214	293	84	93	338	43	31	0,73	0,63
18	8	8	221	118	50	40	71	131	59	56	111	123	54	59	0,55	0,46
9	6	8	161	70	118	86	110	158	106	106	52	79	47	51	0,41	0,47
2	14	16	78	160	182	138	44	117	197	62	26	35	61	59	0,66	0,52
12	9	5	95	78	73	59	82	119	108	143	113	104	62	66	0,36	0,48
22	21	23	200	138	56	38	62	59	72	39	89	91	34	52	0,39	0,47
11	13	7	116	260	75	135	92	494	92	13	37	30	32	9	0,33	0,35
18	16	19	257	76	79	167	88	141	92	79	98	67	174	64	0,23	0,41
15	18	14	421	294	310	200	117	108	47	54	20	13	27	41	0,45	0,39
11	9	8	767	236	63	66	62	94	23	25	58	91	223	190	0,32	0,30
14	20	15	181	71	74	62	143	174	93	108	96	74	22	20	0,41	0,31
10	24	15	171	90	62	120	99	183	176	214	71	51	30	17	0,38	0,45
18	15	22	121	101	69	60	60	48	61	57	54	54	48	48	0,49	0,67
11	11	6	107	87	126	74	116	228	109	113	49	52	28	26	0,20	0,36
10	21	15	466	358	72	150	66	65	40	36	88	28	64	51	0,57	0,54
19	23	23	369	126	55	63	74	170	56	55	120	110	41	31	0,73	0,60
10	9	7	386	110	57	45	121	108	46	55	59	69	34	40	0,23	0,35
9	13	13	131	50	66	40	80	128	56	69	52	64	35	38	0,42	0,63
18	10	17	116	45	75	45	94	107	88	170	47	51	34	34	0,49	0,33
10	13	17	152	61	104	38	86	237	57	84	40	59	26	20	0,41	0,44
7	12	9	107	71	136	141	38	194	134	108	49	30	53	28	0,34	0,28
18	7	4	251	103	122	78	58	110	40	69	47	61	42	50	0,38	0,39
11	11	16	244	154	104	89	88	171	79	85	68	58	35	26	0,59	0,58
19	6	10	215	127	70	33	67	102	72	66	38	45	32	39	0,42	0,51
8	7	11	220	70	77	51	190	157	180	172	110	119	18	26	0,44	0,29
15	17	24	329	117	80	46	67	114	38	59	46	41	31	33	0,29	0,38
20	16	9	138	84	54	127	102	269	342	215	88	72	22	19	0,32	0,20
10	21	16	341	141	92	70	122	147	47	71	65	86	30	30	0,36	0,41
4	7	10	150	172	104	123	118	91	230	69	110	114	47	57	0,46	0,33
26	22	17	218	194	87	71	67	70	93	42	81	47	64	86	0,39	0,41
8	7	7	209	92	91	90	81	115	90	96	30	56	42	45	0,50	0,44
11	18	7	196	148	92	87	361	253	91	64	69	78	10	17	0,21	0,51
15	10	19	98	36	43	79	133	51	66	81	37	49	22	28	0,38	0,22

10	18	16	93	110	48	47	68	203	101	63	105	95	46	26	0,22	0,47
17	11	18	122	101	47	174	73	108	82	97	51	32	22	27	0,25	0,27
22	11	18	173	65	82	56	189	383	67	63	114	186	13	13	0,36	0,26
11	14	15	255	96	111	105	95	91	117	91	61	52	44	88	0,42	0,39
23	16	10	225	118	63	53	116	143	57	54	49	60	40	51	0,58	0,50
11	19	13	232	217	92	160	131	108	51	39	70	60	167	204	0,42	0,35
17	12	8	387	203	48	60	81	105	66	36	74	73	30	46	0,40	0,34
18	12	8	268	118	99	126	75	174	63	79	143	108	70	39	0,27	0,25
15	21	19	312	128	44	126	100	106	39	71	63	38	27	42	0,40	0,46
19	8	6	236	100	109	70	57	109	46	79	45	66	42	49	0,34	0,33
7	10	14	116	67	100	80	163	164	53	112	85	75	21	22	0,18	0,36
6	25	16	186	88	117	123	214	250	123	71	41	40	15	15	0,19	0,21
21	19	25	118	52	35	50	37	118	108	107	51	56	31	26	0,24	0,37
23	8	6	190	64	42	35	60	78	74	104	58	91	54	94	0,33	0,33
9	8	8	261	119	87	103	84	84	37	21	65	75	198	251	0,29	0,30
11	17	12	105	48	290	109	163	217	130	80	15	34	23	17	0,35	0,27
12	19	14	59	44	81	63	117	214	94	104	95	95	26	46	0,53	0,36
20	23	5	154	786	116	129	70	113	48	17	49	62	36	48	0,53	0,47
11	21	14	276	124	76	67	118	174	77	140	46	90	27	21	0,46	0,30
14	8	3	93	37	39	30	51	94	70	115	83	91	69	78	0,54	0,22
12	9	8	164	194	87	93	72	91	57	51	54	45	40	53	0,33	0,42
18	12	19	121	90	59	66	60	49	59	60	57	54	53	51	0,52	0,64
5	4	5	132	57	74	94	85	166	102	103	91	71	57	45	0,65	0,55
13	11	6	397	167	59	42	54	123	39	45	86	88	60	37	0,33	0,43
11	10	7	143	109	63	27	77	120	145	37	35	60	26	26	0,35	0,31
15	8	7	139	113	67	109	116	159	110	93	85	51	21	27	0,83	0,51
10	13	18	79	60	58	79	103	86	115	89	48	41	37	34	0,67	0,58
10	8	4	116	56	42	21	70	148	61	68	50	67	34	38	0,37	0,41
10	9	7	153	46	41	39	67	283	103	134	52	80	39	17	0,44	0,40
5	9	16	168	84	103	82	84	91	75	62	52	75	41	39	1,64	0,43
23	17	21	135	121	89	50	33	42	78	56	75	87	62	50	0,40	0,56
18	17	10	191	121	47	58	55	23	46	62	50	36	46	48	0,25	0,42
12	23	15	107	80	86	63	87	80	131	154	51	69	26	19	0,38	0,32
17	20	16	286	132	52	79	69	152	73	46	63	59	32	35	0,52	0,47
7	21	17	150	84	66	74	75	38	64	115	74	89	48	47	0,32	0,29
14	18	16	111	100	78	81	54	70	71	61	47	45	39	46	0,43	0,47
14	16	15	600	162	78	88	60	145	21	90	45	42	48	27	0,42	0,48
5	7	6	132	166	48	102	138	135	85	58	74	76	47	41	0,26	0,56
13	8	10	130	76	89	80	95	145	66	70	38	37	36	40	0,37	0,37
18	12	13	245	131	218	56	39	139	57	52	15	54	33	35	0,35	0,27
21	23	21	163	118	38	49	37	31	68	41	68	59	46	42	0,44	0,43
9	12	10	227	86	33	41	36	80	83	50	240	123	51	45	0,40	0,48
6	14	10	391	109	361	539	153	683	66	58	27	20	35	7	0,28	0,25
19	21	17	216	188	118	71	71	101	68	55	55	64	47	40	0,42	0,36
17	5	8	98	72	73	115	74	137	84	70	41	38	63	43	0,24	0,42
7	20	17	171	107	129	300	87	94	103	114	53	35	56	53	0,39	0,38
15	13	14	139	44	168	57	86	97	48	81	20	39	28	27	0,54	0,36
16	14	21	124	103	80	68	60	50	84	64	53	53	50	54	0,52	0,69
17	14	12	52	47	43	40	37	168	148	133	74	79	48	28	0,45	0,41
16	6	5	242	100	45	30	29	111	63	56	80	67	58	62	0,43	0,32
8	14	16	203	254	97	56	78	91	71	27	62	71	41	35	0,45	0,33
9	12	15	162	98	92	54	77	156	77	46	64	67	35	21	0,52	0,61
16	11	19	86	63	45	44	63	88	91	74	50	57	54	45	0,40	0,63

21	8	11	75	39	40	67	34	111	110	50	43	40	45	36	0,30	0,25
16	18	15	232	98	76	150	277	92	66	93	85	19	20	32	0,33	0,29
12	13	18	260	54	54	47	48	100	39	70	62	57	44	43	0,35	0,44
11	12	14	54	21	47	70	23	63	145	73	58	75	67	67	0,34	0,32
16	7	8	147	118	67	64	37	70	61	62	104	85	53	55	0,38	0,39

5									
C3 R	C3 L	C4 R	C4 L	C5 R	C5 L	C6 R	C6 L	C7 R	C7 L
0,39	0,38	0,32	0,22	0,25	0,28	0,27	0,46	0,24	0,46
0,24	0,44	0,58	0,45	0,49	0,45	0,30	0,24	2,85	2,96
0,44	0,46	0,33	0,39	0,39	0,20	0,35	0,28	0,59	0,45
0,44	0,42	0,29	0,36	0,36	0,34	0,13	0,18	0,24	0,18
0,41	0,25	0,48	0,36	0,27	0,21	0,33	0,50	0,48	0,41
0,11	0,09	0,34	0,49	0,20	0,37	0,36	0,42	0,26	0,23
0,25	0,34	0,35	0,29	0,34	0,61	0,21	0,31	0,34	0,29
0,65	0,54	0,33	0,64	0,43	0,37	0,25	0,28	0,49	0,37
0,51	0,69	0,44	0,30	0,30	0,24	0,22	0,27	0,38	0,46
0,17	0,26	0,21	0,30	0,48	0,60	0,34	0,30	0,30	0,42
0,39	0,55	0,46	0,33	0,31	0,38	0,20	0,30	0,42	0,45
0,21	0,17	0,28	0,31	0,40	0,37	0,31	0,30	0,47	0,47
0,21	0,20	0,40	0,27	0,67	0,65	0,42	0,34	0,45	0,47
0,23	0,27	0,34	0,23	0,21	0,27	0,37	0,41	0,42	0,27
0,15	0,21	0,41	0,36	0,42	0,48	0,25	0,50	0,42	0,34
0,45	0,36	0,39	0,41	0,34	0,36	0,44	0,36	0,36	0,38
0,13	0,20	0,50	0,47	0,22	0,25	0,23	0,28	0,33	0,21
0,24	0,36	0,23	0,25	0,50	0,28	0,27	0,31	0,32	0,33
0,18	0,16	0,36	0,34	0,32	0,49	0,25	0,42	0,32	0,39
0,38	0,38	0,44	0,38	0,32	0,29	0,46	0,43	0,32	0,28
0,12	0,18	0,24	0,24	0,35	0,24	0,46	0,26	0,51	0,43
0,24	0,38	0,32	0,32	0,18	0,22	0,28	0,28	0,30	0,36
0,35	0,42	0,29	0,40	0,43	0,32	0,43	0,53	0,59	0,47
0,27	0,31	0,31	0,29	0,28	0,60	0,48	0,53	0,51	0,06
0,00	0,44	0,32	0,29	0,22	0,36	0,25	0,43	0,45	0,46
0,65	0,33	0,40	0,50	0,39	0,45	0,31	0,20	0,33	0,20
0,45	0,48	0,62	0,61	0,32	0,35	0,34	0,41	0,34	0,78
0,45	0,26	0,22	0,25	0,35	0,39	0,25	0,32	0,52	0,39
0,37	0,24	0,42	0,42	0,29	0,28	0,28	0,35	0,33	0,41
0,61	0,44	0,51	0,57	0,49	0,46	0,28	0,46	0,38	0,36
0,34	0,31	0,33	0,44	0,24	0,38	0,23	0,38	0,25	0,41
0,69	0,78	0,22	0,21	0,22	0,17	0,31	0,35	0,32	0,49
0,42	0,51	0,20	0,52	0,44	0,20	0,40	0,50	0,48	0,43
0,26	0,27	0,34	0,43	0,24	0,39	0,62	0,41	0,27	0,32
0,42	0,32	0,37	0,24	0,19	0,38	0,24	0,25	0,38	0,32
0,29	0,44	0,74	0,35	0,42	0,40	0,38	0,57	0,57	0,34

0,32	0,32	0,30	0,28	0,46	0,35	0,19	0,23	0,25	0,27
0,30	0,25	0,45	0,22	0,29	0,34	0,46	0,35	0,50	0,35
0,34	0,34	0,15	0,26	0,36	0,39	0,28	0,53	0,21	0,45
0,47	0,53	0,58	0,23	0,37	0,44	0,46	0,32	0,54	0,62
0,28	0,12	0,36	0,38	0,35	0,50	0,46	0,43	0,49	0,34
0,35	0,30	0,31	0,47	0,27	0,26	0,35	0,34	0,52	0,51
0,44	0,49	0,57	0,62	0,28	0,32	0,56	0,57	0,68	0,57
0,43	0,44	0,43	0,40	0,36	0,47	0,42	0,45	0,48	0,39
0,26	0,77	0,41	0,59	0,44	0,32	0,25	0,24	0,41	0,47
0,38	0,47	0,39	0,45	0,63	0,67	0,34	0,45	0,50	0,64
0,42	0,42	0,60	0,43	0,48	0,28	0,31	0,33	0,69	0,67
0,55	0,48	0,20	0,34	0,24	0,30	0,45	0,32	0,42	0,43
0,36	0,27	0,72	0,42	0,22	0,31	0,48	0,50	0,50	0,48
0,39	0,44	0,54	0,45	0,28	0,55	0,36	0,45	0,37	0,47
0,00	0,66	0,38	0,42	0,39	0,34	0,48	0,41	0,44	0,49
0,47	0,41	0,41	0,59	0,23	0,43	0,47	0,49	0,75	0,53
0,54	0,40	0,45	0,42	0,44	0,56	0,34	0,36	0,58	0,63
0,50	0,45	0,41	0,46	0,75	0,40	0,55	0,39	0,56	0,90
0,52	0,42	0,23	0,27	0,30	0,66	0,37	0,33	0,40	0,39
0,38	0,00	0,41	0,33	0,23	0,29	0,36	0,40	0,44	0,38
0,37	0,15	0,47	0,57	0,22	0,41	0,45	0,43	0,43	0,46
0,31	0,35	0,38	0,40	0,52	0,52	0,30	0,17	0,65	0,65
0,35	0,34	0,37	0,37	0,43	0,39	0,44	0,43	0,38	0,43
0,48	0,40	0,49	0,48	0,34	0,36	0,46	0,29	0,47	0,46
0,36	0,58	0,19	0,37	0,18	0,27	0,30	0,44	0,44	0,42
0,22	0,00	0,27	0,41	0,23	0,32	0,51	0,45	0,56	0,47
0,43	0,40	0,55	0,35	0,14	0,22	0,42	0,40	0,41	0,51
0,73	0,73	0,70	0,68	0,25	0,44	0,32	0,46	0,38	0,42
0,54	0,51	0,62	0,38	0,52	0,37	0,44	0,34	0,39	0,42
0,34	0,48	0,41	0,45	0,41	0,41	0,28	0,23	0,31	0,52
0,22	0,22	0,24	0,33	0,28	0,30	0,53	0,45	0,39	0,56
0,52	0,82	0,39	0,37	0,16	0,38	0,22	0,33	0,35	0,43
0,29	0,29	0,43	0,40	0,39	0,46	0,34	0,43	0,45	0,45
0,41	0,33	0,22	0,31	0,31	0,23	0,28	0,22	0,38	0,28
0,35	0,41	0,33	0,33	0,34	0,27	0,34	0,35	0,40	0,42
0,28	0,27	0,31	0,31	0,29	0,28	0,60	0,48	0,53	0,51
0,39	0,29	0,36	0,28	0,21	0,30	0,49	0,37	0,45	0,36
0,28	0,37	0,30	0,24	0,25	0,37	0,47	0,29	0,48	0,50
0,34	0,24	0,58	0,51	0,41	0,32	0,36	0,46	0,54	0,42
0,21	0,30	0,60	0,32	0,51	0,41	0,17	0,30	0,47	0,30
0,00	0,44	0,32	0,29	0,22	0,36	0,25	0,43	0,45	0,46
0,75	0,71	0,46	0,57	0,57	0,70	0,40	0,50	0,70	0,91
0,49	0,39	0,32	0,46	0,50	0,50	0,39	0,39	0,53	0,45
0,46	0,51	0,30	0,34	0,35	0,46	0,38	0,40	0,58	0,51
0,36	0,38	0,20	0,32	0,45	0,42	0,35	0,38	0,52	0,45
0,40	0,20	0,38	0,55	0,34	0,32	0,20	0,32	0,53	0,40
0,37	0,27	0,34	0,51	0,27	0,40	0,20	0,11	0,34	0,35
0,43	0,40	0,42	0,61	0,31	0,51	0,37	0,38	0,42	0,50
0,60	0,41	0,54	0,45	0,39	0,33	0,41	0,40	0,63	0,65
0,09	0,35	0,25	0,38	0,15	0,31	0,51	0,49	0,48	0,41
0,34	0,35	0,20	0,20	0,41	0,40	0,26	0,34	0,51	0,62
0,57	0,31	0,28	0,54	0,39	0,23	0,45	0,52	0,43	0,37
0,44	0,50	0,36	0,37	0,46	0,45	0,37	0,35	0,66	0,68

0,30	0,28	0,00	3,20	0,16	0,22	0,34	0,44	0,27	0,57
0,28	0,61	0,47	0,45	0,28	0,27	0,20	0,61	0,50	0,59
0,34	0,48	0,47	0,40	0,34	0,44	0,22	0,32	0,61	0,65
0,29	0,00	0,00	0,50	0,45	0,34	0,28	0,32	0,52	0,40
0,50	0,50	0,19	0,43	0,69	0,56	0,40	0,50	0,84	0,84
0,61	0,40	0,56	0,44	0,39	0,23	0,43	0,49	0,96	0,96
0,47	0,31	0,41	0,51	0,37	0,37	0,27	0,40	0,26	0,65
0,51	0,20	0,52	0,44	0,20	0,40	0,50	0,48	0,43	0,43
0,65	0,38	0,30	0,35	0,53	0,35	0,45	0,34	0,39	0,35
0,31	0,51	0,67	0,76	0,28	0,38	0,48	0,46	0,46	0,46
0,41	0,24	0,57	0,44	0,33	0,39	0,49	0,44	0,40	0,40
0,50	0,40	0,46	0,44	0,39	0,45	0,43	0,46	0,60	0,62
0,76	0,43	0,56	0,27	0,42	0,25	0,57	0,53	0,52	0,56
0,45	0,34	0,41	0,43	0,26	0,37	0,48	0,36	0,32	0,32
0,20	0,25	0,42	0,31	0,36	0,45	0,20	0,29	0,43	0,37
0,00	0,24	0,57	0,19	0,24	0,36	0,56	0,44	0,46	0,57
0,78	0,22	0,21	0,22	0,17	0,31	0,35	0,32	0,49	0,45
0,00	0,39	0,54	0,28	0,30	0,29	0,45	0,58	0,55	0,60
0,28	0,64	0,43	0,27	0,27	0,24	0,35	0,36	0,57	0,67
0,46	0,43	0,22	0,50	0,36	0,46	0,48	0,71	0,36	0,45
0,66	0,48	0,45	0,39	0,10	0,72	0,51	0,43	0,41	0,49
0,57	0,33	0,42	0,41	0,49	0,42	0,29	0,24	0,57	0,54
0,43	0,44	0,43	0,46	0,55	0,39	0,49	0,55	0,50	0,31
0,37	0,52	0,29	0,41	0,25	0,49	0,33	0,38	0,45	0,43
0,48	0,49	0,36	0,37	0,26	0,09	0,19	0,52	0,43	0,49
0,20	0,33	0,21	0,38	0,59	0,41	0,20	0,24	0,31	0,46
0,25	0,34	0,33	0,23	0,15	0,34	0,54	0,34	0,29	0,34
0,36	0,22	0,72	0,66	0,39	0,38	0,35	0,35	0,41	0,49
0,50	0,39	0,41	0,47	0,34	0,49	0,51	0,57	0,49	0,46
0,19	0,28	0,39	0,37	0,27	0,34	0,40	0,31	1,43	1,43
0,38	0,38	0,25	0,25	0,27	0,36	0,35	0,35	0,28	0,36
0,71	0,43	0,53	0,81	0,59	0,43	0,35	0,28	0,44	0,67
0,41	0,42	0,52	0,62	0,36	0,56	0,49	0,59	0,22	0,42
0,32	0,43	0,51	0,65	0,56	0,53	0,37	0,39	0,60	0,62
0,10	0,34	0,55	0,44	0,36	0,27	0,38	0,34	0,43	0,39
0,61	0,61	0,33	0,31	0,33	0,29	0,65	0,65	0,86	0,86
0,23	0,29	0,46	0,49	0,29	0,26	0,37	0,43	0,42	0,45
0,39	0,69	0,52	0,40	0,26	0,55	0,37	0,63	0,61	0,47
0,44	0,46	0,48	0,48	0,36	0,42	0,71	0,71	0,49	0,63
0,42	0,39	0,40	0,30	0,52	0,51	0,76	0,48	0,24	0,37
0,27	0,20	0,33	0,41	0,32	0,34	0,45	0,49	0,32	0,26
0,75	0,65	0,18	0,30	0,28	0,26	0,24	0,34	0,60	0,58
0,41	0,45	0,41	0,21	0,31	0,32	0,46	0,33	0,49	0,51
0,40	0,51	0,36	0,45	0,48	0,44	0,44	0,39	0,59	0,60
0,33	0,38	0,26	0,25	0,25	0,28	0,34	0,22	0,37	0,46
0,44	0,59	0,41	0,49	0,47	0,65	0,49	0,40	0,41	0,52
0,52	0,43	0,36	0,31	0,26	0,30	0,31	0,31	0,42	0,40
0,65	0,49	0,51	0,65	0,59	0,49	0,52	0,19	0,32	0,64
0,46	0,48	0,31	0,33	0,43	0,42	0,20	0,26	0,30	0,21
0,35	0,27	0,28	0,38	0,35	0,26	0,23	0,37	0,41	0,48
0,47	0,49	0,55	0,59	0,32	0,32	0,39	0,18	0,16	0,21
0,29	0,29	0,27	0,41	0,20	0,29	0,33	0,34	0,35	0,30
0,47	0,77	0,35	0,47	0,45	0,45	0,51	0,51	0,31	0,31

0,60	0,60	0,28	16,00	0,33	0,41	0,19	0,30	0,67	0,39
0,39	0,18	0,46	0,31	0,24	0,20	0,36	0,18	0,38	0,37
0,29	0,39	0,39	0,31	0,78	0,78	0,31	0,31	0,36	0,36
0,65	0,24	0,47	0,61	0,32	1,39	0,38	0,27	0,37	0,63
0,50	0,67	0,56	0,53	0,27	0,38	0,43	0,41	0,32	0,27
0,35	0,30	0,31	0,46	0,31	0,37	0,28	0,41	0,37	0,89
0,53	0,37	0,64	0,55	0,36	0,40	0,39	0,33	0,73	0,42
0,31	0,77	0,23	0,36	0,45	0,46	0,23	0,45	0,37	0,39
0,54	0,32	0,34	0,21	0,47	0,34	0,23	0,21	0,47	0,62
0,71	0,38	0,22	0,30	0,27	0,24	0,24	0,29	0,55	0,55
0,42	0,65	0,33	0,37	0,31	0,42	0,29	0,42	0,48	0,23
0,22	0,45	0,33	0,33	0,40	0,34	0,17	0,31	0,31	0,28
0,23	0,24	0,25	0,29	0,43	0,37	0,23	0,21	0,42	0,45
0,67	0,70	0,41	0,41	0,35	0,31	0,41	0,24	0,48	0,32
0,28	0,32	0,38	0,41	0,37	0,42	0,51	0,47	0,62	0,53
0,33	0,72	0,34	0,34	0,42	0,34	0,34	0,46	0,21	0,18
0,28	0,25	0,32	0,37	0,38	0,29	0,41	0,54	0,37	0,47
0,36	0,39	0,32	0,31	0,31	0,40	0,27	0,42	0,33	0,41
0,46	0,34	0,43	0,67	0,49	0,42	0,45	0,32	0,42	0,65
0,49	0,49	0,29	0,46	0,22	0,29	0,19	0,31	0,40	0,27
0,48	0,62	0,42	0,34	0,36	0,30	0,34	0,33	0,52	0,30
0,48	0,48	0,52	0,59	0,43	0,62	0,53	0,62	0,33	0,39
0,76	0,46	0,85	0,83	0,36	0,76	0,33	0,35	0,71	0,55
0,33	0,35	0,46	0,44	0,43	0,47	0,19	0,23	0,34	0,55
0,21	0,27	0,59	0,18	0,15	0,24	0,19	0,45	0,53	0,51
0,55	0,32	0,38	0,37	0,46	0,47	0,33	0,26	0,58	0,49
0,37	0,37	0,53	0,33	0,39	0,34	0,48	0,46	0,44	0,49
0,71	0,51	0,24	0,25	0,37	0,23	0,37	0,34	0,54	0,47
0,51	0,38	0,45	0,43	0,15	0,26	0,38	0,24	0,40	0,27
0,30	0,55	0,48	0,58	0,34	0,48	0,54	0,37	0,55	0,48
0,11	0,42	0,39	0,40	0,22	0,26	0,36	0,41	0,37	0,42
0,37	0,20	0,42	0,45	0,20	0,31	0,24	0,41	0,30	0,38
0,39	0,37	0,49	0,19	0,34	0,29	0,36	0,36	0,46	0,46
0,32	0,55	0,33	0,23	0,19	0,14	0,31	0,43	0,35	0,36
0,43	0,44	0,46	0,46	0,61	0,53	0,45	0,48	0,58	0,42
0,59	0,55	0,42	0,47	0,20	0,39	0,58	0,35	0,50	0,46
0,36	0,36	0,34	0,47	0,39	0,55	0,21	0,32	0,34	0,24
0,45	0,63	0,45	0,45	0,39	0,30	0,85	0,56	0,48	0,45
0,18	0,35	0,13	0,51	0,95	0,26	0,52	0,46	0,54	0,76
0,19	0,24	0,30	0,37	0,40	0,25	0,17	0,25	0,56	0,41
0,54	0,46	0,29	0,55	0,43	0,59	0,33	0,30	0,38	0,38
0,46	0,33	0,34	0,33	0,39	0,42	0,28	0,27	0,52	0,61
0,24	0,24	0,36	0,27	0,39	0,36	0,22	0,26	0,20	0,55
0,47	0,50	0,32	0,56	0,52	0,27	0,43	0,46	0,41	0,35
0,24	0,24	0,42	0,51	0,45	0,57	0,46	0,51	0,58	0,53
0,23	0,25	0,41	0,46	0,45	0,35	0,29	0,35	0,60	0,55
1,14	0,34	0,19	0,39	0,40	0,26	0,37	0,50	0,72	0,58
0,39	0,40	0,46	0,53	0,42	0,57	0,52	0,59	0,25	0,39
0,53	0,46	0,35	0,65	0,54	0,37	0,34	0,58	0,46	0,53
0,43	0,23	0,31	0,50	0,42	0,39	0,15	0,37	0,34	0,29
0,44	0,46	0,48	0,39	0,21	0,31	0,26	0,32	0,44	0,42
0,46	0,54	0,28	0,40	0,38	0,33	0,28	0,42	0,24	0,32
0,73	0,90	0,51	0,58	0,23	0,52	0,69	0,65	0,49	0,55

0,22	0,19	0,38	0,32	0,25	0,33	0,39	0,33	0,43	0,49
0,38	0,38	0,21	0,57	0,42	0,33	0,39	0,36	0,42	0,59
0,56	0,58	0,41	0,33	0,25	0,33	0,27	0,46	0,49	0,43
0,24	0,19	0,41	0,41	0,22	0,29	0,33	0,35	0,43	0,39
0,30	0,54	0,34	0,27	0,25	0,36	0,32	0,16	0,34	0,42
